# Whole-Brain Afferent Inputs to the Caudate Nucleus, Putamen, and Accumbens Nucleus in the Tree Shrew Striatum

**DOI:** 10.3389/fnana.2021.763298

**Published:** 2021-11-02

**Authors:** Rong-Jun Ni, Yu-Mian Shu, Tao Li, Jiang-Ning Zhou

**Affiliations:** ^1^Mental Health Center and Psychiatric Laboratory, West China Hospital of Sichuan University, Chengdu, China; ^2^Huaxi Brain Research Center, West China Hospital of Sichuan University, Chengdu, China; ^3^School of Architecture and Civil Engineering, Chengdu University, Chengdu, China; ^4^Chinese Academy of Sciences Key Laboratory of Brain Function and Diseases, School of Life Sciences, University of Science and Technology of China, Hefei, China

**Keywords:** caudate nucleus, putamen, accumbens nucleus, thalamus, neural circuits

## Abstract

Day-active tree shrews have a well-developed internal capsule (ic) that clearly separates the caudate nucleus (Cd) and putamen (Pu). The striatum consists of the Cd, ic, Pu, and accumbens nucleus (Acb). Here, we characterized the cytoarchitecture of the striatum and the whole-brain inputs to the Cd, Pu, and Acb in tree shrews by using immunohistochemistry and the retrograde tracer Fluoro-Gold (FG). Our data show the distribution patterns of parvalbumin (PV), nitric oxide synthase (NOS), calretinin (CR), and tyrosine hydroxylase (TH) immunoreactivity in the striatum of tree shrews, which were different from those observed in rats. The Cd and Pu mainly received inputs from the thalamus, motor cortex, somatosensory cortex, subthalamic nucleus, substantia nigra, and other cortical and subcortical regions, whereas the Acb primarily received inputs from the anterior olfactory nucleus, claustrum, infralimbic cortex, thalamus, raphe nucleus, parabrachial nucleus, ventral tegmental area, and so on. The Cd, Pu, and Acb received inputs from different neuronal populations in the ipsilateral (60, 67, and 63 brain regions, respectively) and contralateral (23, 20, and 36 brain regions, respectively) brain hemispheres. Overall, we demonstrate that there are species differences between tree shrews and rats in the density of PV, NOS, CR, and TH immunoreactivity in the striatum. Additionally, we mapped for the first time the distribution of whole-brain input neurons projecting to the striatum of tree shrews with FG injected into the Cd, Pu, and Acb. The similarities and differences in their brain-wide input patterns may provide new insights into the diverse functions of the striatal subregions.

## Introduction

The striatum is composed of the ventral and dorsal striatum (Ni et al., 2018). The ventral striatum contains the accumbens nucleus (Acb). In humans and primates, the dorsal striatum is subdivided into the caudate nucleus (Cd), internal capsule (ic), and putamen (Pu) (Provost et al., 2015). A well-developed ic clearly separates the Cd and Pu in primates and humans, whereas the underdeveloped ic is scattered in most regions of the rostral and middle parts of the dorsal striatum in rodents (Ni et al., 2018). The striatum exhibits functional heterogeneity. The Cd is important in many functions, such as motor response (Kesner and Gilbert, 2006), motor skill learning (Choi et al., 2020), and psychiatric illnesses (Vostrikov and Uranova, 2020). The Pu seems to participate in motor learning (Liebrand et al., 2020), auditory responses (Salisbury et al., 2021), cocaine addiction (Ersche et al., 2021), and psychiatric disorders (Kolomeets and Uranova, 2020). In addition, the Acb is involved in motor control (Sawada et al., 2015), reward processing (Muench et al., 2018), motivation (Baumgartner et al., 2021), addiction (Crofton et al., 2021), mental disorders (Terrillion et al., 2017), and social defeat stress (Yin et al., 2021).

The day-active tree shrew is the phylogenetically closest living relative of primates (Fan et al., 2013). Previous studies have reported that the tree shrew is closer to primates than to rodents in terms of brain evolution, such as the neocortex (Römer et al., 2018), hippocampus (Ni et al., 2015), thalamus (Maher et al., 2021), cerebellum (Sillitoe et al., 2004), and striatum (Rice et al., 2011; Ni et al., 2018). Furthermore, a previous paper reported that the ic separates the dorsal striatum into the Cd and Pu in tree shrews (Ni et al., 2018), which is similar to the structure in primates (Holt et al., 1997; Cragg et al., 2002; Rice et al., 2011). The use of non-human primates in medical research is costly and time-consuming, and ethical concerns and restrictions should also be considered (Zhang et al., 2014; Yao, 2017). Although rodents are widely used in biomedical research, the findings are sometimes difficult to interpret due to species disparities and other reasons (Yao, 2017). These studies indicate that the tree shrew is a potential model for human striatal disorders, including Parkinson’s disease (Ma et al., 2013).

The morphological characteristics of the Cd, Pu, and Acb in tree shrews have been described previously by immunohistochemical analysis of parvalbumin (PV), tyrosine hydroxylase (TH), calbindin, acetylcholinesterase, calretinin (CR), somatostatin, and neuropeptide Y (Rice et al., 2011; Yamashita et al., 2012; McCollum and Roberts, 2014; Ni et al., 2015, 2018). A previous paper indicated that there is a marked distinction in the distribution of PV-immunoreactive (-ir) cells in the striatum of tree shrews compared to that of rats (Ni et al., 2018). Cytoarchitectonic characterization of the ventral and dorsal striatum in tree shrews has been presented previously (Rice et al., 2011; Ni et al., 2018); however, differences in the distribution of TH-, nitric oxide synthase (NOS)-, and CR-ir neurons between tree shrews and rats have not been investigated.

The striatum receives and integrates information from afferent neurons in the rhinencephalon, telencephalon, diencephalon, mesencephalon, metencephalon, and myelencephalon before passing this information to the substantia nigra pars reticulata (forming the direct pathway) and to the external globus pallidus (forming the indirect pathway) (Smith and Bolam, 1991; Gandia et al., 1993; Shammah-Lagnado et al., 1996; Tsumori et al., 2000; Barry et al., 2018). The striatum has a critical role in motor control, motor skills learning, movement execution, and action selection (Rubia et al., 1999; Bailey and Mair, 2006; Durieux et al., 2012; Park et al., 2020). Previous studies have documented that the striatum in tree shrews receives inputs from the thalamic nuclei, pulvinar, and medial geniculate body (Lin et al., 1984; Day-Brown et al., 2010). Additionally, previous findings by anterograde and retrograde tracing methods have shown that both the central nucleus (Pc) and the dorsal nucleus (Pd) of the pulvinar project to the Cd and Pu (Day-Brown et al., 2010). However, the input patterns for the Cd, Pu, and Acb in the tree shrew brain have not been examined at the whole-brain level.

In this study, we assessed the expression of PV, NOS, CR, and TH in the striatum of tree shrews (*Tupaia belangeri chinensis*). Furthermore, we compared the distributions of these proteins in the Cd, ic, Pu, and Acb of tree shrews with those in rats. Additionally, to investigate the characteristics of the brain-wide inputs to the striatal subregions, including the Cd, Pu, and Acb, we injected Fluoro-Gold (FG) into the subregions of the striatum to label projection cells retrogradely. Finally, we provide whole-brain maps of afferent projections to the subregions of the striatum in tree shrews.

## Materials and Methods

### Animals

Twelve adult male Chinese tree shrews (*Tupaia belangeri chinensis*) (from the breeding colony at the Animal House Center of the Kunming Institute of Zoology, Kunming, China) and five Sprague-Dawley rats (3 months of age) were used. The tree shrews and rats were housed in animal facilities under a 12-h light/dark cycle (lights on at 8:00 a.m.), with food and water available *ad libitum*. All protocols in the present study were approved by the Animal Care and Use Committees of the Sichuan University and the University of Science and Technology of China. All efforts were made to minimize animal suffering as well as to reduce the number of animals used.

### Surgery

Adult male tree shrews (*n* = 6, 8–12 months of age) were deeply anesthetized using pentobarbital sodium (80 mg/kg, i.p.). The head of the tree shrew was fixed in a digital stereotaxic frame (Stoelting Company, United States). The incisor bar was adjusted until the heights of the lambda and bregma skull points were equal to achieve a flat skull position. At this position, the incisor bar of the stereotaxic apparatus was decreased to 5.0 mm below the horizontal zero plane. A sagittal incision was cut along the midline of the tree shrew head. A glass micropipette (outer diameter, approximately 50 μm) was filled with 2% FG solution (Fluorochrome, Denver, CO, United States) and connected to a 10-μl Hamilton syringe that was driven by an UltraMicroPump (Model: UMP3; World Precision Instruments, Inc. Sarasota, FL, United States) equipped with a Micro4 MicroSyringe Pump Controller (Model: UMC4; World Precision Instruments, Inc., Sarasota, FL, United States). For retrograde labeling of striatum-projecting neurons, unilateral pressure injections of 2% FG solution were injected into different striatal regions in volumes of 80 nl using the following coordinates taken from the tree shrew brain atlas (Zhou and Ni, 2016): Cd (anteroposterior: +8.15 mm from interaural line, mediolateral: –2.60 mm from midline, dorsoventral: –3.65 mm from dura), Pu (anteroposterior: + 8.15 mm from interaural line, mediolateral: –4.40 mm from midline, dorsoventral: –5.60 mm from dura), and Acb (anteroposterior: +9.85 mm from interaural line, mediolateral: –1.90 mm from midline, dorsoventral: –7.50 mm from dura). At the end of each injection, the syringe needle was left in place for an additional 10 min, and all tree shrews were allowed to survive for 7 days.

The FG is one of the most popular fluorescent tracers used for retrograde labeling in neuroscience studies (Brog et al., 1993; Comoli et al., 2012; Bácskai et al., 2014; Dopeso-Reyes et al., 2014; Scheel et al., 2020). Labeling efficacy of the FG tracer has been extensively studied (Richmond et al., 1994; Novikova et al., 1997; Puigdellívol-Sánchez et al., 1998, 2002; Zele et al., 2010).

### Tissue Preparation

All tree shrews and rats were deeply anesthetized with pentobarbital sodium (80 mg/kg, i.p.), and perfused with 0.9% saline followed by 4% paraformaldehyde (PFA) in phosphate buffer (PB, 0.1 M, pH 7.4). After perfusion with 4% PFA, the brains were removed and postfixed by immersion in the same fixative overnight at 4°C. Then, the tree shrew and rat brains were soaked in 15% sucrose in phosphate-buffered saline (PBS: 0.1 M; pH 7.4) until the tissues sank to the bottom of the container and then immersed in 30% sucrose in PBS until they sank (3–5 days). Thereafter, the tissues were frozen and sectioned in the coronal or sagittal planes at 40 μm on a Leica microtome (Leica CM1950, Germany). All sections were stored in cryoprotectant solution at –20°C until use. Six tree shrews with the most accurate tracer delivery sites were used to map brain-wide afferent inputs to Cd, Pu, and Acb in the coronal plane.

### Green-Fluorescent Nissl Staining

A series of whole-brain sections of FG-treated tree shrews (at 240-μm intervals) were incubated with diluted NeuroTrace^TM^ 500/525 (1:100; Invitrogen, United States) green-fluorescent Nissl stains for 30 min at 37°C. Sections were then washed in PBS before mounting on glass slides in a 0.5% gelatin solution. Finally, immunofluorescent sections were scanned using Imager. Z2 (Carl Zeiss, Germany) with an automated acquisition system (TissueFAXS Plus, TissueGnostics GmbH, Austria).

### Immunohistochemistry and Immunofluorescence for Fluoro-Gold

Another series of adjacent sections was taken from FG-treated tree shrews (at 320-μm intervals). These sections were used for immunohistochemistry, as previously described (Ni et al., 2016). Briefly, the free-floating sections were rinsed and treated with 0.3% hydrogen peroxide and 0.5% Triton X-100 in PBS to quench endogenous peroxidase activity. Then, after incubation with 5% normal goat serum (Solarbio, Beijing, China) in PBST (PBS containing 0.5% Triton X-100), the free-floating sections were immunoreacted with primary antibodies of rabbit anti-FG (Fluorochrome Cat# 52-9600, RRID:AB_2314408, at 1:400 dilution) in PBST containing 5% normal goat serum at 4°C for 3 days. Signal amplification was performed with biotinylated goat anti-rabbit IgG (1:200; Vector Laboratories, Burlingame, CA, United States) and avidin-biotin peroxidase complex (1:200; Vector Laboratories). Subsequent chromogen development was performed with 0.05% diaminobenzidine (Sigma-Aldrich). The sections were mounted on gelatin-coated glasses and allowed to air-dry overnight before being dehydrated in ethanol-xylene and coverslipped using neutral quick drying glue. Photographs were taken using Imager.Z2 (Carl Zeiss, Germany) with an automated acquisition system (TissueFAXS Plus, TissueGnostics GmbH, Austria).

For the simultaneous detection of FG for tracing analysis, FG-labeled sections containing the compact part of the substantia nigra (SNC) were incubated with mouse monoclonal anti-TH (Millipore, MAB5280, at 1:2000 dilution). Native fluorescence was used to identify the FG (blue). Subsequently, the sections were incubated with Alexa Fluor^®^ 594-AffiniPure donkey anti-mouse IgG (H + L) (Jackson ImmunoResearch, 715-585-151, at 1:400 dilution) at 37°C for 1 h. Finally, the sections were incubated with diluted NeuroTrace^TM^ 500/525 (1:100; Invitrogen, United States) green-fluorescent Nissl stains for 30 min at 37°C. Sections were then washed in PBS before mounting on glass slides in a 0.5% gelatin solution. Photographs were taken using Imager.Z2 (Carl Zeiss, Germany).

### Immunohistochemistry for Parvalbumin, Nitric Oxide Synthase, Calretinin, and Tyrosine Hydroxylase

Four series of free-floating sections were taken at the level of the striatum of tree shrews and rats. These free-floating sections were processed for immunohistochemical detection of PV, NOS, CR, and TH and subjected to the same procedure as above with the following exceptions. The free-floating sections were incubated with primary antibodies against mouse monoclonal anti-PV (Millipore Cat# MAB1572, RRID:AB_2174013, at 1:2000 dilution), rabbit anti-NOS antibody (Millipore Cat# AB5380, RRID:AB_91824, at 1:2000 dilution), mouse monoclonal anti-CR (Millipore Cat# MAB1568, RRID:AB_94259, at 1:2000 dilution), and mouse monoclonal anti-TH (Millipore Cat# MAB5280, RRID:AB_2201526, at 1:2000 dilution) in PBST containing 5% normal goat serum overnight at 4°C. Amplification was performed with biotinylated horse anti-mouse (1:200; Vector Laboratories) and goat anti-rabbit (1:200; Vector Laboratories) secondary antibodies, followed by incubation in avidin-biotin peroxidase complex (1:200; Vector Laboratories) and diaminobenzidine with hydrogen peroxide as the chromogen.

Another series of sections were subjected to PV, NOS, CR, or TH immunoperoxidase labeling and then stained with a 0.02% thionin acetate salt solution for 20 min. Nissl-stained sections were then quickly rinsed in PBS, dehydrated in ethanol-xylene, coverslipped using neutral quick drying glue, and visualized using a whole slide scanner (TissueFAXS Plus, TissueGnostics GmbH, Austria).

### Digital Photomicrographs and Analysis

All photomicrographs were taken using an automated acquisition system (TissueFAXS Plus, TissueGnostics GmbH, Austria). All digital photomicrographs were adjusted for image size, cropping, and brightness/contrast using Adobe Photoshop CS6 (Adobe Systems, United States). Neuroanatomical localization and designation of the striatum and other brain regions were based on previously published brain atlases of the tree shrew (Zhou and Ni, 2016) and the rat (Paxinos and Watson, 2007). A series of representative coronal sections from FG-injected tree shrews were selected from one or two tree shrew brains. All camera lucida drawings and plots were edited using Adobe Illustrator (Adobe Systems, United States) by tracing the digital photomicrographs of representative sections. Every individual inset in Figures is based on tracing the single section.

For quantitative analysis of the density of PV-ir, NOS-ir, and CR-ir profiles in the striatum of tree shrews and rats, more than three representative sections from each animal (tree shrews: *n* = 6; rats: *n* = 5) were chosen for counting. The density of PV-ir, NOS-ir, and CR-ir profiles was measured at 200 × total magnification. Automatic cell counting in ImageJ was used to analyze these representative sections based on previous studies (Grishagin, 2015; Georgina et al., 2021). All processes were assembled as scripts in the macros (ImageJ) as follows. The RGB images were converted to 8-bit gray scale image (Image→8-bit). Pre-processing of images was done through the Band-pass filter (Image → Process → FFT → Bandpass filter). The attributes in the Bandpass filter were set to filter large = 10 pixels, filter small = 3 pixels, suppression stripes were set to “none,” tolerance of direction was set at 5% and autoscale was set to “Saturate image when autoscaling.” The threshold function was then used to binarize the images (Image → Adjust → Threshold), where the RenyiEntropy threshold was selected and set threshold (50, 155) was specified. The final step was the counting of the positive profiles using the “analyze particles” tool with size = 50–150, circularity = 0.30–1.00, and show = Bare Outlines (Analyze → Analyze Particles). In ImageJ, a random-offset grid (500 μm × 500 μm) was applied to each representative section. Only the grids in the region of interest were counted for each section. The photomicrographs were numbered, and the assessment was performed by two observers blinded to the group assignment. The density of PV-ir, NOS-ir, and CR-ir profiles was determined from the number of positive profiles per grid. The data obtained from all grids in each animal were pooled.

The intensity of TH-ir fibers in the striatum of tree shrews and rats was assessed by the semi-automated quantification of fiber densities in ImageJ (Grider et al., 2006). All processes were assembled as scripts in the macros (ImageJ) as follows. The RGB images were converted to 8-bit gray scale image (Image→8-bit). Then images were applied the Hessian filter of FeatureJ with the largest eigenvalues of Hessian tensor, not absolute eigenvalue comparison, and smoothing scale = 0.5 (Analyze → Plugins → FeatureJ → FeatureJ Hessian). The resulting 8-bit gray scale images were converted into a binary image by applying a threshold (“auto” setting for threshold). In ImageJ, a random-offset grid (500 μm × 500 μm) was applied to each representative section. Only the grids in the region of interest were analyzed for each section. The corresponding value of TH-ir fibers in the corpus callosum (cc) was also analyzed for each image. These were then normalized to the value obtained from the grids in the region of interest. Normalized data obtained from all grids in each animal were pooled.

### Statistics

All data are presented as the mean ± SEM, and no data were excluded. For normally distributed data, the significance of differences among the mean values for the obtained data of various subregions was calculated by the unpaired two-tailed *t*-test and one-way ANOVA (Tukey’s test was used for *post hoc* comparison) using SPSS 19 (SPSS Inc., Chicago, IL, United States). In all cases, a probability value of *p* < 0.05 was considered statistically significant. The graphs were plotted using GraphPad Prism (GraphPad Software, San Diego, CA, United States).

## Results

### Expression of Parvalbumin, Nitric Oxide Synthase, Calretinin, and Tyrosine Hydroxylase in the Striatum of Tree Shrews and Rats

**FIGURE 1:**
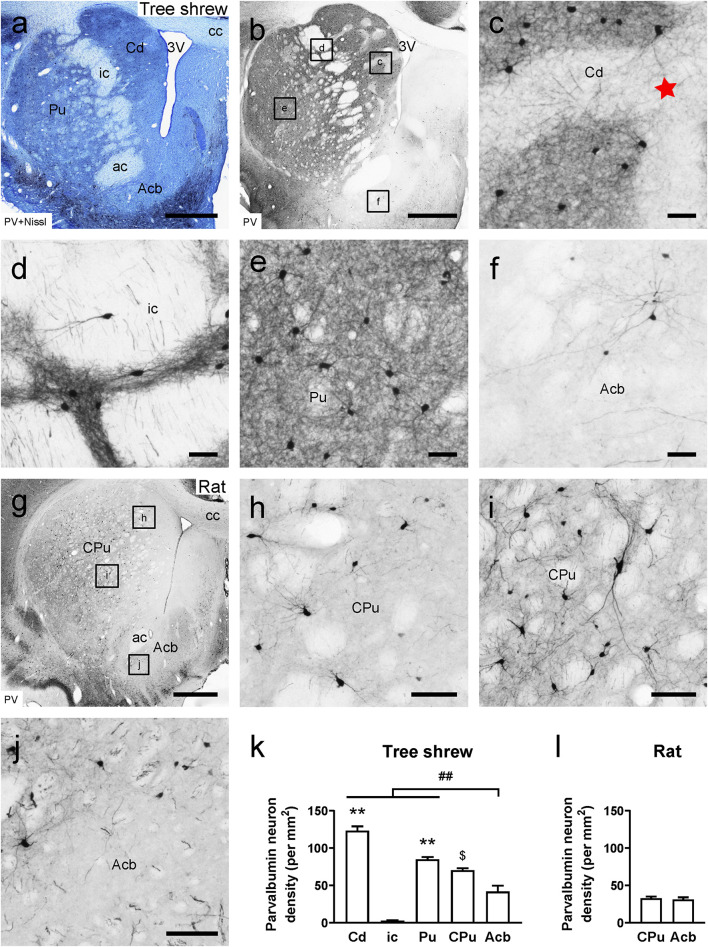
Photomicrographs of parvalbumin (PV)-ir staining are shown in the striatum of the tree shrew and rat brains. **(a)** Photomicrograph showing Nissl staining (blue) following PV immunoperoxidase labeling (black) in a coronal section of the tree shrew striatum. **(b)** Low-magnification photomicrograph illustrating PV-ir staining in an adjacent section of the tree shrew striatum. **(c–f)** High-magnification image of PV immunoreactivity in the caudate nucleus (Cd), internal capsule (ic), putamen (Pu), and accumbens nucleus (Acb) of the tree shrew striatum (boxed area in b). The red star indicates the location of the striosome and matrix compartments in the Cd. **(g)** Low-magnification photomicrograph illustrating PV-ir staining in an equivalent section of the rat striatum. **(h–j)** High-magnification photomicrograph illustrating PV immunoreactivity in the caudate putamen (CPu) and Acb of the rat striatum (boxed area in g). **(k)** Quantitative analysis of the density of PV-ir cells in distinct subregions of the striatum in the tree shrew. **(l)** Quantitative analysis of the density of PV-ir cells in the CPu and Acb of the rat striatum. Values are expressed as the mean ± SEM. **P < 0.01 compared with the ic; ##*P* < 0.01 compared with the Cd, ic, and Pu; $*P* < 0.05 compared with the Acb. Scale bars = 1000 μm in **(a,b)**, g; 50 μm in **(c–f)**; 100 μm in **(h–j)**.

In the tree shrew striatum, PV immunoreactivity was found in the Cd, ic, Pu, and Acb (Figures 1a,b,e). A high density of PV-ir cells and fibers was observed in the Cd, whereas quite a few PV-ir neurons were present in the striosome and matrix compartments of the Cd (Figures 1b,c). Sparse PV-ir fibers were scattered in the striosome and matrix compartments (Figure 1c). Similarly, sparse PV-ir neurons and fibers were distributed in the ic (Figure 1d). Moderate numbers of PV-ir cells and fibers were located in the Acb of tree shrews (Figure 1f). In the rat striatum, a moderate density of PV-ir cells and fibers was seen in the CPu and Acb (Figures 1g–j). In the striatum of tree shrews, analysis of variance showed highly significant differences in the density of PV-ir cells among the Cd, ci, Pu, CPu, and Acb (*F*_4, 25_ = 95.813, *P* < 0.001; Figure 1k). A much larger number of PV-ir cells were found in the Cd (123.43 ± 5.579) and Pu (85.05 ± 3.076) than in the ic (2.94 ± 0.653; Figure 1k). In addition, a slightly lower number of PV-ir neurons were observed in the Acb (41.99 ± 7.656) compared with that in the Cd and Pu (Figure 1k). However, the density of PV-ir cells in the CPu (32.90 ± 2.100) of rats was similar to that in the Acb (31.29 ± 2.785; *t*_8_ = 0.460, *P* > 0.05; Figure 1l).

**FIGURE 2:**
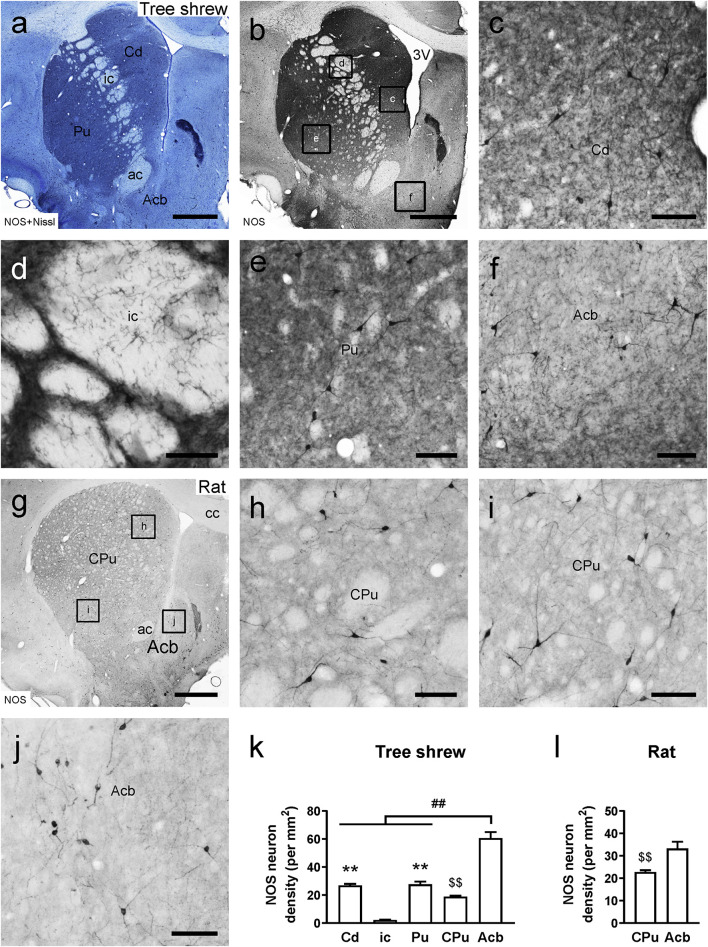
Comparison of nitric oxide synthase (NOS)-ir staining in the striatum of the tree shrew and rat brains. **(a)** Photomicrograph showing Nissl staining (blue) following NOS immunoperoxidase labeling (black) in a coronal section of the tree shrew striatum. **(b)** Low-magnification photomicrograph illustrating NOS-ir staining in an adjacent section of the tree shrew striatum. **(c–f)** High-magnification image of NOS immunoreactivity in the caudate nucleus (Cd), internal capsule (ic), putamen (Pu), and accumbens nucleus (Acb) of the striatum (boxed area in b). **(g)** Low-magnification photomicrograph illustrating NOS immunoreactivity in an equivalent section of the rat striatum. **(h–j)** High-magnification image of NOS immunoreactivity in the caudate putamen (CPu) and Acb of the rat striatum (boxed area in **g**). **(k)** Comparison of the density of NOS-ir neurons in distinct subregions of the striatum in the tree shrew. **(l)** Comparison of the density of NOS-ir cells in the CPu and Acb of the rat striatum. Values are expressed as the mean ± SEM. **P < 0.01 compared with the ic; ##*P* < 0.01 compared with the Cd, ic, and Pu; $$*P* < 0.01 compared with the Acb. Scale bars = 1000 μm in **(a,b,g)**; 100 μm in **(c–f,h–j)**.

Adjacent sections of the tree shrew striatum were stained with thionin solution following NOS immunoperoxidase labeling to provide neurochemical anatomy (Figures 2a,b). A moderate density of NOS-ir cells and fibers was visualized in the Cd and Pu (Figures 2c,e). Conversely, few NOS-ir cells and fibers were found in the ic (Figure 2d). In addition, there were darkly stained NOS-ir neurons and fibers in the Acb (Figure 2f). In the rat striatum, the CPu contained a moderate density of NOS-ir cells and fibers, whereas in the Acb a high density of NOS-ir cells was present (Figures 2g–j). In the tree shrew striatum, analysis of variance showed highly significant differences in the density of NOS-ir cells among the Cd (26.97 ± 1.031), ci (2.28 ± 0.237), Pu (27.67 ± 1.982), CPu (18.97 ± 0.530), and Acb (60.69 ± 4.271; *F*_4_,_25_ = 96.039, *P* < 0.001; Figure 2k). The lowest density of NOS-ir cells was detected in the ic, whereas the highest density of positive cells was located in the Acb. Similarly, in the rat striatum, the Acb (33.29 ± 3.010) contained a higher density of NOS-ir cells than the CPu (22.77 ± 0.834; *t*_8_ = –3.365, *P* < 0.01; Figure 2l).

**FIGURE 3:**
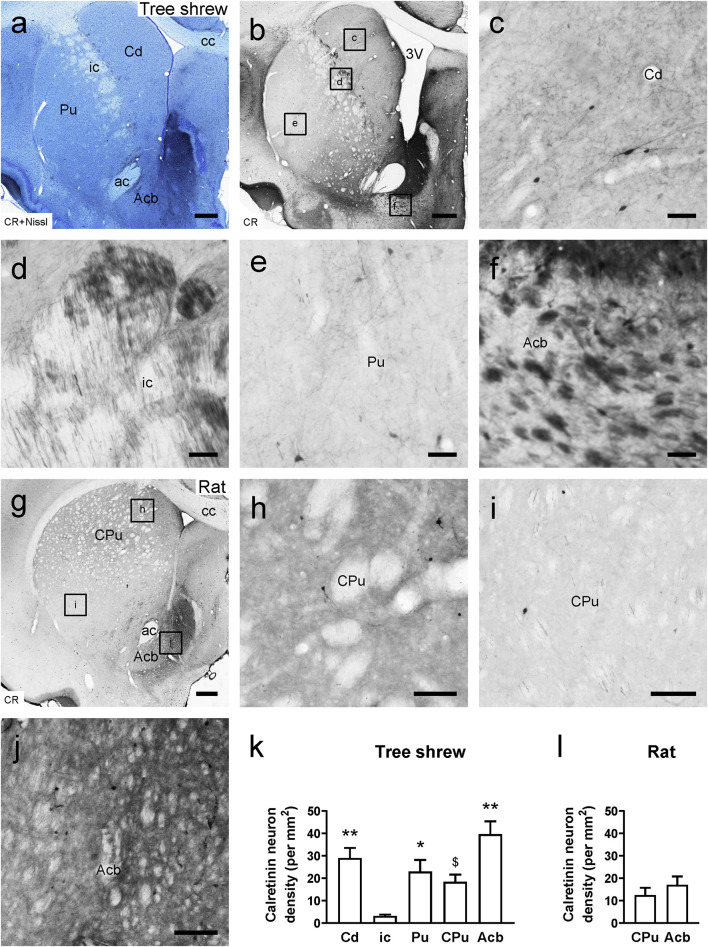
Bright-field photomicrographs of calretinin (CR) immunoreactivity are shown in the tree shrew and rat striata. **(a)** Low-magnification photomicrograph showing Nissl staining (blue) following CR-ir staining (black) in the tree shrew striatum. **(b)** Low-magnification photomicrograph illustrating CR immunoreactivity in an adjacent section of the striatum. **(c–f)** High-magnification image of CR-ir neurons and fibers in the caudate nucleus (Cd), internal capsule (ic), putamen (Pu), and accumbens nucleus (Acb) of the striatum (boxed area in **b**). **(g)** Low-magnification photomicrograph illustrating CR immunoreactivity in an equivalent section of the rat striatum. **(h–j)** High-magnification image of CR immunoreactivity in the caudate putamen (CPu) and Acb of the rat striatum (boxed area in **g**). **(k)** Comparison of the density of CR-ir neurons in distinct subregions of the striatum in the tree shrew. **(l)** Comparison of the density of CR-ir cells in the CPu and Acb of the rat striatum. Values are expressed as the mean ± SEM. *P < 0.05, **P < 0.01 compared with the ic; $*P* < 0.05 compared with the Acb. Scale bars = 500 μm in **(a,b,g)**; 50 μm in **(c–f)**; 100 μm in **(h–j)**.

In the tree shrew brain, neuronal cell bodies and processes exhibiting CR immunostaining were observed throughout the striatum (Figures 3a,b). A moderate number of CR-ir cells were scattered in the Cd, Pu, and Acb, whereas few CR-ir cells were present in the ic (Figures 3c–f). Sparsely and weakly stained CR-ir fibers were seen in the Cd and Pu. However, densely and intensely stained fibers were observed in the dorsal but not the ventral part of the ic (Figure 3d). Additionally, densely and intensely stained CR-ir fibers were found in the medial or dorsal but not in the lateral or ventral part of the Acb (Figures 3a,b,f). In the rat brain, sparsely and weakly stained CR-ir neurons and fibers were distributed throughout the dorsal striatum (Figures 3g–i). Similar to the tree shrew Acb, the rat Acb contained densely and intensely stained fibers in the medial and dorsal parts but not in the lateral and ventral parts (Figures 3g,j). In the tree shrew striatum, analysis of variance showed highly significant differences in the density of CR-ir cells among the Cd (29.06 ± 4.464), ci (3.19 ± 0.602), Pu (22.97 ± 5.224), CPu (18.41 ± 3.241), and Acb (39.62 ± 5.765; *F*_4_,_25_ = 9.941, *P* < 0.001; Figure 3k). The lowest density of CR-ir cells was detected in the ic, whereas the highest density of positive cells was located in the Acb. However, in the rat striatum, there was no significant difference in the density of CR-ir cells between the CPu and Acb (Figure 3l).

**FIGURE 4:**
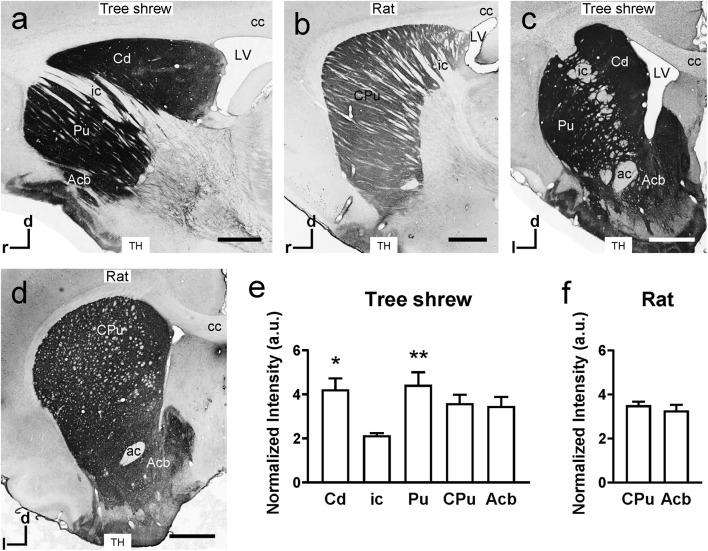
Comparison of tyrosine hydroxylase (TH) immunoreactivity in the striatum of tree shrews and rats. **(a,b)** Bright-field photomicrographs of TH immunoreactivity are shown in sagittal sections of the tree shrew and rat striata. **(c,d)** Low-magnification photomicrograph illustrating TH immunoreactivity in coronal sections of the striatum in tree shrews and rats. **(e)** A graph showing the normalized intensity of TH-ir fibers in the caudate nucleus (Cd), internal capsule (ic), putamen (Pu), and accumbens nucleus (Acb) of tree shrews. **(f)** Comparison of the normalized intensity of TH-ir fibers in the caudate putamen (CPu) and Acb of the rat striatum. Values are expressed as the mean ± SEM. *P < 0.05, **P < 0.01 compared with the ic. Scale bars = 1000 μm in **(a–d)**. ac, anterior commissure; cc, corpus callosum; d, dorsal; l, lateral; LV, lateral ventricle; r, rostral.

In the sagittal sections of the tree shrew brain, there were darkly stained TH-ir fibers in the Cd and Pu but lightly stained fibers in the ic (Figure 4a), which separated the dorsal striatum into the Cd and Pu. However, in rats, the ic was nearly devoid of TH-ir fibers and scattered in the CPu from dorsal to ventral (Figure 4b). In addition, darkly stained TH-ir fibers were present throughout the Acb in the coronal sections from tree shrews and rats (Figures 4c,d). In the tree shrew striatum, analysis of variance showed highly significant differences in the normalized intensity of TH-ir fibers among the Cd (4.22 ± 0.498), ic (2.14 ± 0.094), Pu (4.43 ± 0.569), CPu (3.60 ± 0.382), and Acb (3.47 ± 0.412; *F*_4_,_30_ = 4.485, *P* < 0.01; Figure 4e). The lowest intensity of TH-ir fibers was detected in the ic of tree shrews. However, in the rat striatum, there was no significant difference in the normalized intensity of TH-ir fibers between the CPu (3.51 ± 0.163) and Acb (3.28 ± 0.254; Figure 4f).

### Whole-Brain Mapping of Afferent Projections to the Caudate Nucleus in Tree Shrews

**FIGURE 5:**
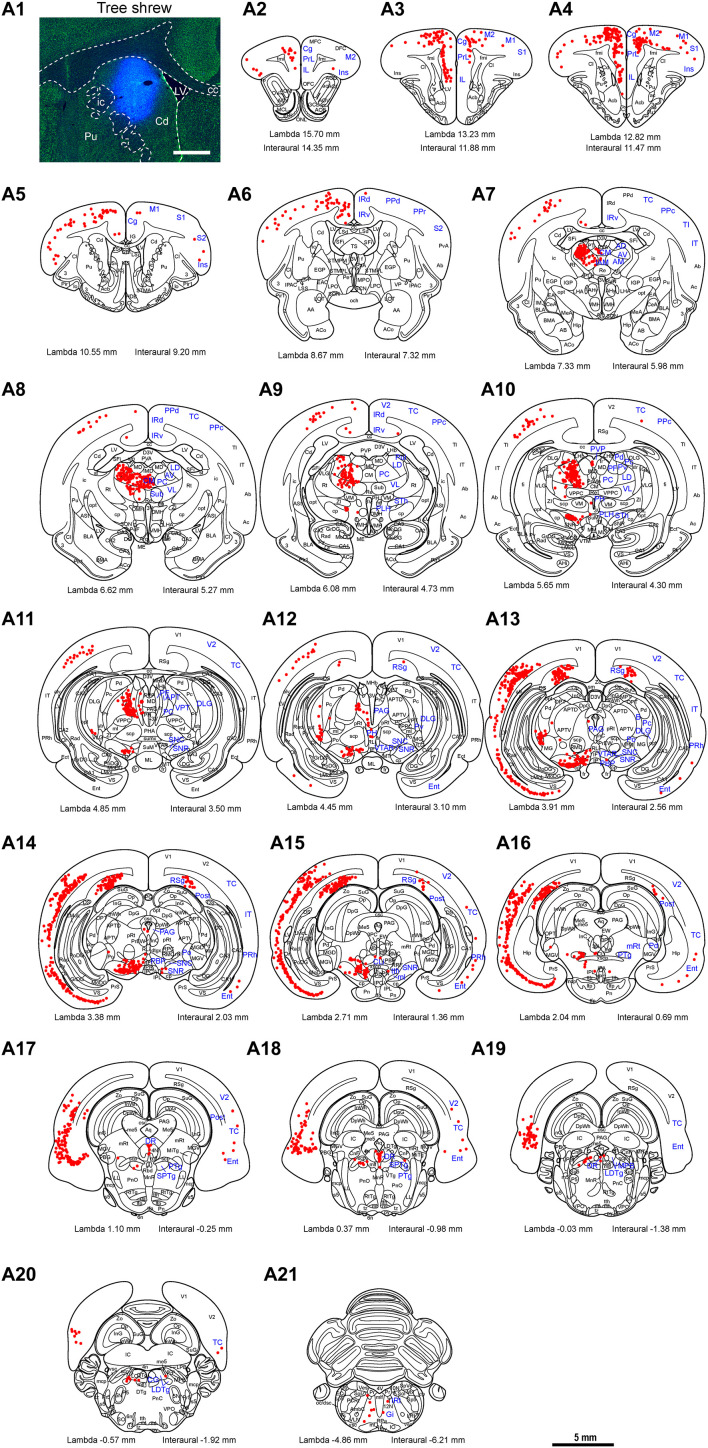
Whole-brain mapping of afferent projections to the caudate nucleus (Cd) in tree shrews. **(A1)** Unilateral pressure injection of Fluoro-Gold (FG) retrograde tracer (blue) into the Cd of the tree shrew brain. The Cd was determined by staining with NeuroTrace^TM^ 500/525-Green Fluorescent Nissl Stain (green). **(A2-A21)** Cd-projecting neurons (red dots) were present in the tree shrew brain (blue letters) from rostral to caudal. Representative coronal sections were selected from two tree shrew brains and referenced to lambda (distance indicated at lower left corner). The density of dots represents the relative density of projecting neurons in those areas. Each dot represents approximately one FG-labeled neuron. For abbreviations, see Abbreviations. Scale bars = 1 mm in **(A1)**; 5 mm in **(A2–A21)**.

**FIGURE 6:**
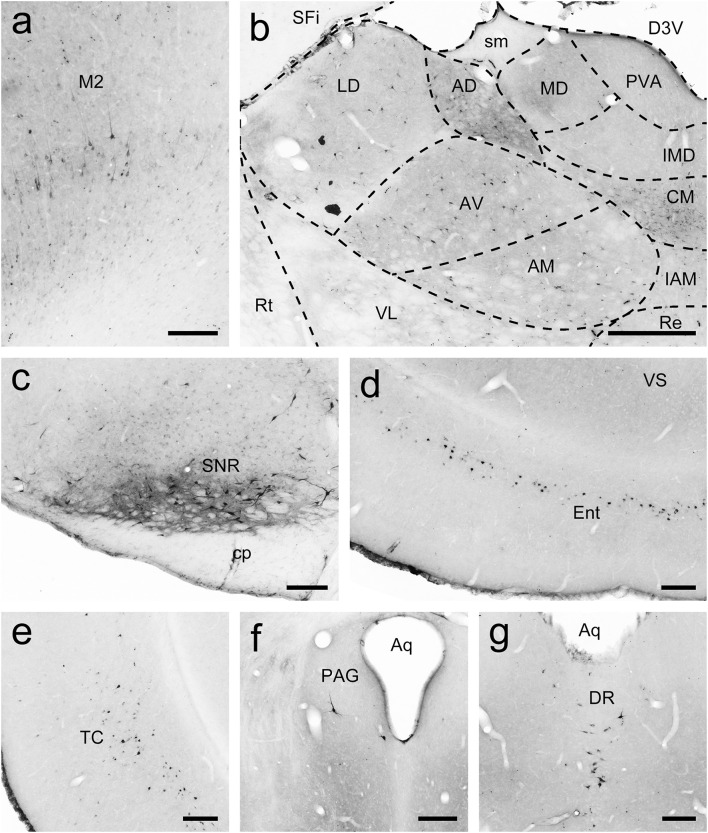
FG-labeled neurons projecting to the caudate nucleus (Cd) were labeled in the primary motor cortex (M1; **a**), thalamus **(b)**, reticular part of the substantia nigra (SNR; **c**), entorhinal cortex (Ent; **d**), temporal cortex (TC; **e**), periaqueductal gray (PAG; **f**), and dorsal raphe nucleus (DR; **g**). The dashed line marks the boundaries between brain regions **(b)**. Scale bars = 200 μm in **(a,c–f)**; 500 μm in **(b)**.

To map the whole-brain afferent inputs to the Cd of tree shrews, the retrograde tracer FG was accurately injected into the Cd (Figure 5A1). The Cd afferent cell populations were located ipsilaterally in the telencephalon, diencephalon, mesencephalon, metencephalon, and myelencephalon of the tree shrew brain from rostral to caudal (Figures 5A2–A21). In the telencephalon, a moderate number of retrogradely labeled cells were observed in the secondary motor cortex (M2), entorhinal cortex (Ent), temporal cortex (TC), and retrosplenial granular cortex (RSg; Figures 5A15, 6a,d,e). A small population of retrogradely labeled cells was distributed in other regions of the neocortex (Figure 5). Some labeled neurons were also observed on the contralateral side, including the insular cortex (Ins), primary somatosensory cortex (S1), secondary somatosensory cortex (S2), primary motor cortex (M1), M2, cingulate cortex (Cg), prelimbic cortex (PrL), infraradiata dorsalis (IRd), RSg, and Ent (Figure 5). In the diencephalon, the different densities of Cd-projecting cells were mainly seen in the thalamus and subthalamic nucleus (Figure 5). A large number of FG-labeled cells were present in the central medial thalamic nucleus (CM), parafascicular thalamic nucleus (PF), and subthalamic nucleus (STh; Figures 5A8,A10,A11, 6b). In addition, a sparse to moderate density of retrogradely labeled cells was scattered in the anteromedial thalamic nucleus (AM), anterior pretectal nucleus (APT), anteroventral thalamic nucleus (AV), dorsal lateral geniculate nucleus (DLG), laterodorsal thalamic nucleus (LD), paracentral thalamic nucleus (PC), Pc, Pd, posterior complex of the thalamus (Po), pulvinar nuclei (Pul), ventral nucleus of the pulvinar (Pv), posterior part of the paraventricular thalamic nucleus (PVP), and ventrolateral thalamic nucleus (VL; Figures 5, 6b). In the mesencephalon, a moderate to high density of FG-labeled cells was visualized in the SNC, reticular part of substantia nigra (SNR), dorsal raphe nucleus (DR), and pedunculopontine tegmental nucleus (PTg; Figures 5A13,A16, 6c,g). Sparse Cd-projecting neurons were observed in the central gray (CG), periaqueductal gray (PAG), parabrachial pigmented nucleus of the ventral tegmental area (PBP), subpeduncular tegmental nucleus (SPTg), and rostral part of the ventral tegmental area (VTAR; Figures 5, 6f). In the metencephalon and myelencephalon of the tree shrew brain, a sparse to moderate density of FG-labeled neurons was scattered in the laterodorsal tegmental nucleus (LDTg), medial parabrachial nucleus (MPB), gigantocellular reticular nucleus (Gi), medial lemniscus (ml), and trigeminothalamic tract (tth; Figure 5). These FG-labeled neurons were mainly distributed ipsilateral to the injection site (Figure 5).

### Whole-Brain Mapping of Afferent Projections to the Putamen in Tree Shrews

**FIGURE 7:**
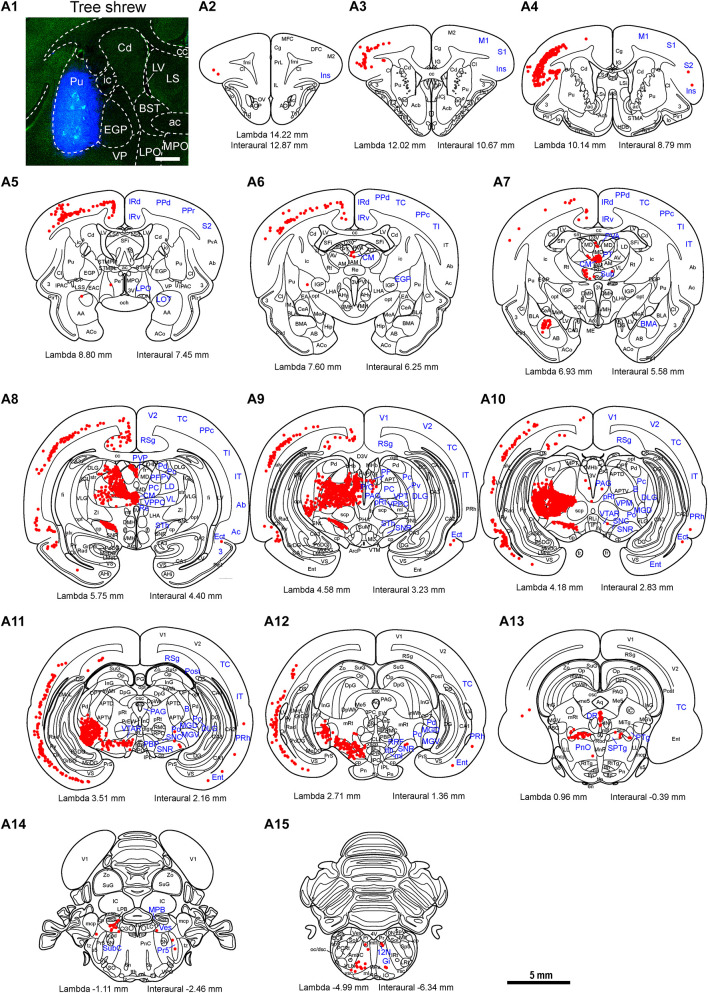
Whole-brain mapping of afferent projections to the putamen (Pu) in tree shrews. **(A1)** Unilateral pressure injection of FG retrograde tracer (blue) into the Pu of the tree shrew brain. The Pu was determined by staining with NeuroTrace^TM^ 500/525-Green Fluorescent Nissl Stain (green). **(A2–A15)** Camera lucida drawings showing the distribution of Pu-projecting neurons (red dots) in the tree shrew brain (blue letters). Representative coronal sections from rostral to caudal were selected from two tree shrew brains and referenced to lambda (distance indicated at lower left corner). The density of dots represents the relative density of projecting neurons in those areas. Each dot represents approximately one FG-labeled neuron. For abbreviations, see Abbreviations. Scale bars = 1 mm in **(A1)**; 5 mm in **(A2–A15)**.

**FIGURE 8:**
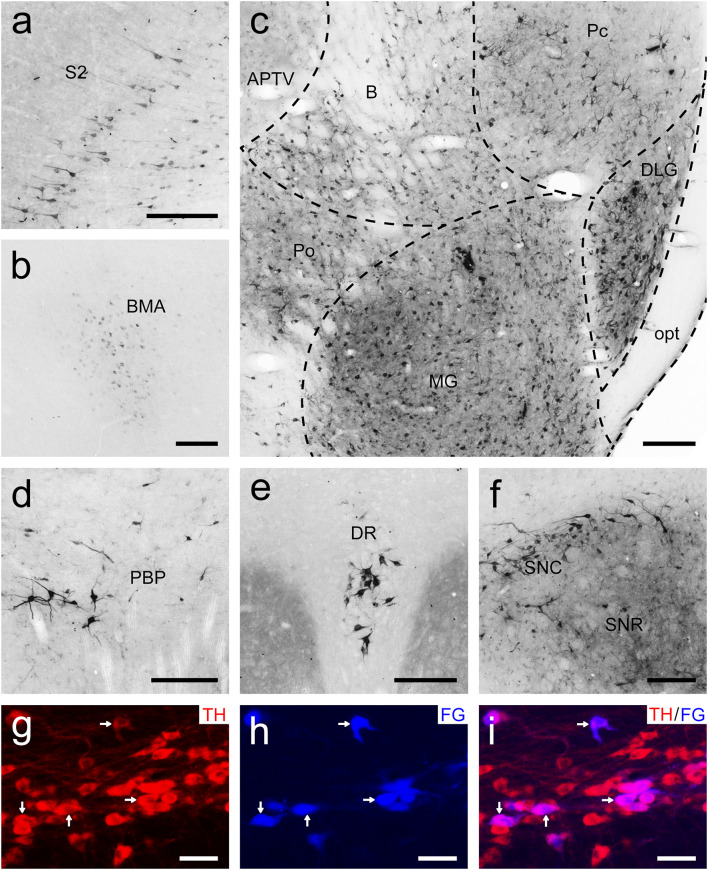
Bright-field photomicrographs showing the distribution of FG-labeled neurons projecting to the putamen (Pu) in the secondary somatosensory cortex (S2; **a**), basomedial amygdaloid nucleus (BMA; **b**), thalamus **(c)**, parabrachial pigmented nucleus of the ventral tegmental area (PBP; **d**), dorsal raphe nucleus (DR; **e**), and substantia nigra **(f)**. The dashed line marks the boundaries between brain regions **(c)**. **(g–i)** Double labeling with anti-tyrosine hydroxylase antibody (TH, red) and FG (blue) in the compact part of the substantia nigra (SNC). The arrows show the double-labeled cells. Scale bars = 200 μm in **(a–f)**; 100 μm in **(g–i)**.

To examine the whole-brain afferent inputs to the Pu, only tree shrews with accurate injections and placements were included in the analyses and data presentation (Figure 7A1). The Pu-projecting neurons were distributed ipsilaterally throughout the tree shrew brain from rostral to caudal (Figures 7A2–A15). In the rhinencephalon, only a few FG-labeled neurons were found in the nucleus of the lateral olfactory tract (LOT; Figure 7A5). In the telencephalon of tree shrews with FG injected into the Pu, most FG-labeled neurons were located ipsilaterally in the S1, S2, posterior parietal rostral area (PPr), and basomedial amygdaloid nucleus (BMA; Figures 7A4,A5,A7, 8a,b). Additionally, a sparse to moderate density of Pu-projecting cells was visible in the Ins, auditory belt area (Ab), auditory core area (Ac), M1, ectorhinal cortex (Ect), Ent, IRd, inferior temporal cortex (IT), posterior parietal caudal area (PPc), perirhinal cortex (PRh), RSg, TC, temporal inferior area (TI), primary visual cortex (V1), and secondary visual cortex (V2; Figures 7A2–A10). In the diencephalon, a large number of FG-labeled neurons were seen in the CM, dorsal part of the medial geniculate nucleus (MGD), ventral part of the medial geniculate nucleus (MGV), PC, PF, PVP, ventral posteromedial thalamic nucleus (VPM), and STh (Figures 7A4,A8–A11, 8c). A small to moderate number of Pu-projecting neurons were observed in the brachium of the superior colliculus (B), DLG, LD, Pc, Pd, Po, precommissural nucleus (PrC), paratenial thalamic nucleus (PT), Pv, anterior part of the paraventricular thalamic nucleus (PVA), reuniens thalamic nucleus (Re), submedius thalamic nucleus (Sub), VL, parvicellular part of the ventral posterior nucleus of the thalamus (VPPC), and ventral posterior thalamic nucleus (VPT; Figures 7A7–A12). In the mesencephalon, a moderate to high number of retrogradely labeled cells were present in the DR, PAG, PBP, p1 reticular formation (pRt), SPTg, PTg, SNR, and SNC (Figures 7A10–A13, 8d–f). In the SNC, some FG-labeled neurons were co-labeled with an antibody against TH (Figures 8g–i). However, a small number of retrogradely labeled cell bodies were found in the retrorubral field (RRF), hypoglossal nucleus (12N), and VTAR (Figures 7A10,A12,A15). In the metencephalon and myelencephalon, only a sparse population of retrogradely FG-labeled cells was present in the MPB, Gi, ml, oral part of the pontine reticular nucleus (PnO), subcoeruleus nucleus (SubC), tth, and vestibular nucleus (Ves; Figures 7A12–A15). The majority of retrogradely FG-labeled neurons were ipsilateral to the injection site throughout the tree shrew brain, whereas the minority were located in the Ect, Ent, PRh, S1, S2, CM, Po, PrC, Re, Sub, 12N, DR, PAG, SPTg, SNR, PTg, VTAR, Gi, principal sensory trigeminal nucleus (Pr5), and Ves (Figure 7).

### Whole-Brain Mapping of Afferent Projections to the Accumbens Nucleus in Tree Shrews

**FIGURE 9:**
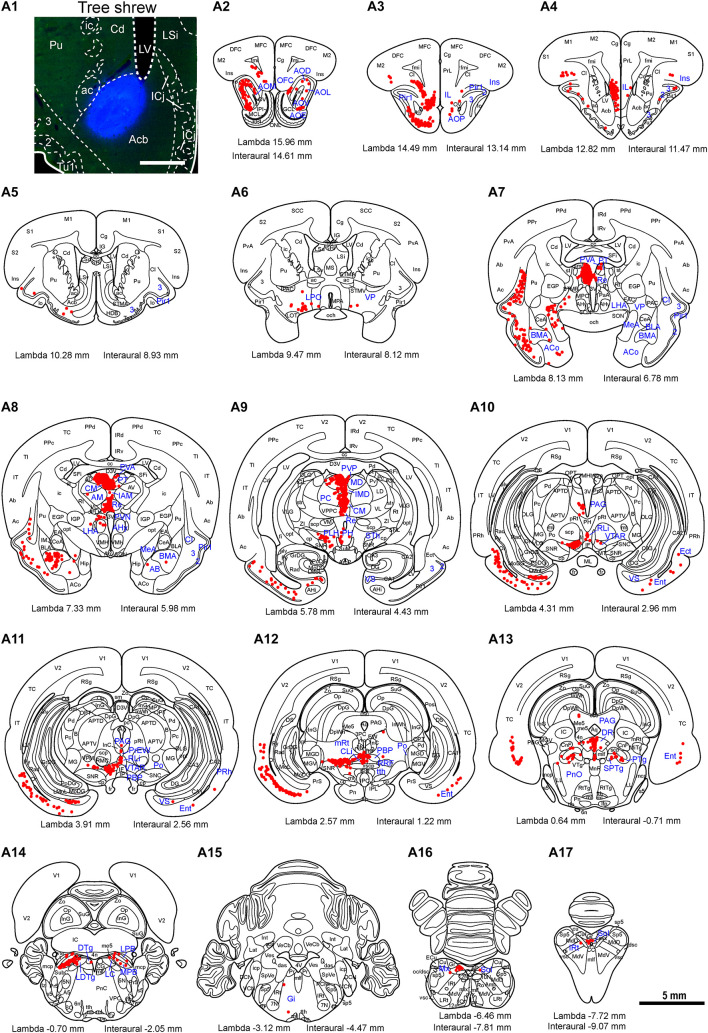
Whole-brain mapping of afferent projections to the accumbens nucleus (Acb) in tree shrews. **(A1)** Photomicrograph showing the injection site of Fluoro-Gold (FG) retrograde tracer in the Acb of the tree shrew brain. The Acb was determined by staining with NeuroTrace 500/525-Green Fluorescent Nissl Stain (green). **(A2–A17)** Acb-projecting neurons (red dots) were present in the tree shrew brain (blue letters) from rostral to caudal. Representative coronal sections were selected from two tree shrew brains and referenced to lambda (distance indicated at lower left corner). The density of dots represents the relative density of projecting neurons in those areas. Each dot represents approximately one FG-labeled neuron. For abbreviations, see Abbreviations. Scale bars = 1 mm in **(A1)**; 5 mm in **(A2–A17)**.

To generate an overall brain-wide distribution of Acb-projecting neurons in tree shrews, we accurately injected the retrograde tracer FG into the Acb (Figure 9A1). Retrogradely FG-labeled cells projecting to the Acb were observed ipsilaterally in the rhinencephalon, telencephalon, diencephalon, mesencephalon, metencephalon, and myelencephalon of the tree shrew brain (Figures 9A2–A17). The most rostrally labeled cells were seen in the anterior olfactory nucleus, whereas the most caudal ones occurred in the solitary nucleus (Sol; Figures 9A2–A17). At the level of the rhinencephalon of tree shrews with FG injected into the Acb, a sparse to moderate number of retrogradely FG-labeled cells were located in the anterior olfactory nucleus, dorsal part (AOD), external part (AOE), lateral part (AOL), medial part (AOM), posterior part (AOP), ventral part (AOV), and layer 3 of the olfactory tubercle (Tu3; Figures 9A2–A5, 10a). At the level of the telencephalon, retrogradely labeled neurons were most concentrated in the claustrum (Cl), infralimbic cortex (IL), and piriform cortex (Figures 9A4,A7, 10b). Additionally, numerous labeled cells were detected in the accessory basal nucleus (AB), anterior cortical amygdaloid nucleus (ACo), basolateral amygdaloid nucleus (BLA), BMA, Ect, Ent, Ins, medial amygdaloid nucleus (MeA), orbital frontal cortex (OFC), ventral pallidum (VP), and ventral subiculum (VS; Figures 9A2–A10, 10c–e). At the level of the diencephalon of tree shrews, the largest population of retrogradely FG-labeled cells was observed in the thalamus, including the Re, PVA, PVP, PT, mediodorsal thalamic nucleus (MD), intermediodorsal thalamic nucleus (IMD), and CM (Figures 9A7–A9, 10f,g). Moreover, a sparse to moderate density of retrogradely labeled cells was clustered in the anterior hypothalamic area (AHy), AM, interanteromedial thalamic nucleus (IAM), PC, posterior hypothalamic nucleus (PH), peduncular part of the lateral hypothalamus (PLH), Po, paraventricular hypothalamic nucleus (PVN), and STh (Figures 9A7–A9). At the level of the mesencephalon, a large population of clustered FG-labeled cells was found ipsilaterally in the caudal linear nucleus of the raphe (CLi), DR, SPTg, rostral linear nucleus of the raphe (RLi), and VTAR (Figures 9A10–A13, 10h,i). In addition, a small population of scattered cells was visible in the mesencephalic reticular formation (mRt), PAG, PBP, pre-Edinger-Westphal nucleus (PrEW), RRF, and PTg (Figures 9A11–A13). At the level of the metencephalon and myelencephalon, a moderate number of retrogradely FG-labeled cells were present in the MPB (Figures 9A14, 10j). Only a sparse population of retrogradely FG-labeled cells was distributed in the dorsal tegmental nucleus (DTg), Gi, intermediate reticular nucleus (IRt), locus coeruleus (LC), LDTg, lateral parabrachial nucleus (LPB), tth, and Sol (Figures 9A14–A17, 10k,l). The FG-labeled neurons were concentrated primarily ipsilateral to the injection site, whereas sparsely labeled neurons were observed in the contralateral hemisphere, including the AOE, AOM, AOP, AOV, AB, BMA, Ect, Ent, layer 2 of the piriform cortex (Pir2), layer 3 of the piriform cortex (Pir3), VP, VS, CM, IAM, IMD, MD, PH, PVA, PVP, Re, CLi, DR, PBP, SPTg, RLi, PTg, VTAR, LC, LPB, MPB, and Sol (Figure 9).

**FIGURE 10:**
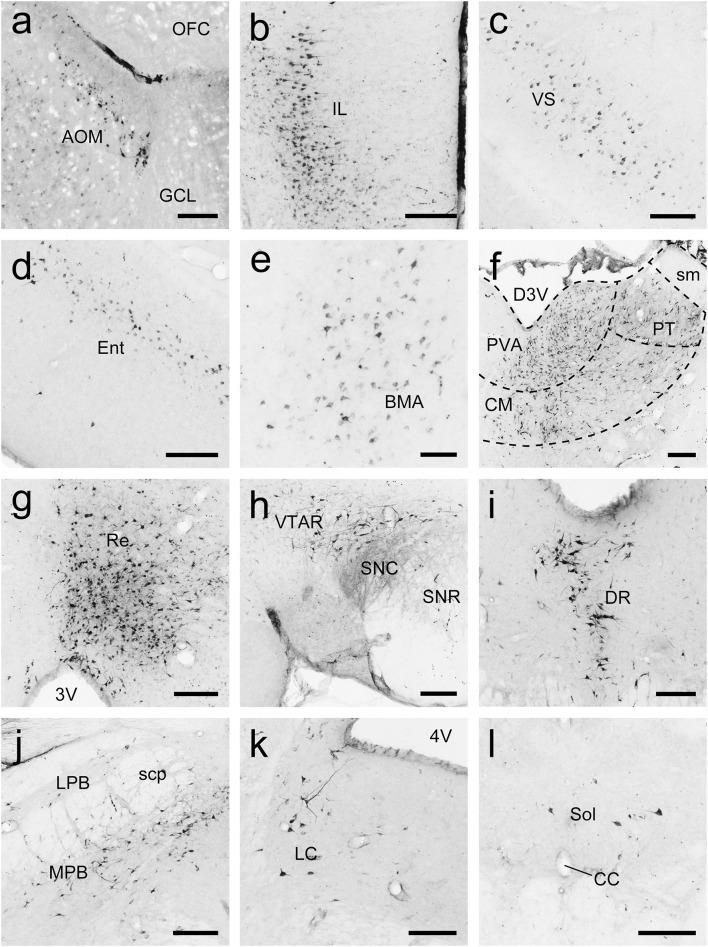
Photomicrographs of coronal sections through the whole brain of tree shrews showing the distribution patterns of FG-labeled neurons projecting to the accumbens nucleus (Acb) in the medial part of the anterior olfactory nucleus (AOM; **a**), infralimbic cortex (IL; **b**), ventral subiculum (VS; **c**), entorhinal cortex (Ent; **d**), basomedial amygdaloid nucleus (BMA; **e**), thalamus **(f)**, reuniens thalamic nucleus (Re; **g**), rostral part of the ventral tegmental area (VTAR; **h**), dorsal raphe nucleus (DR; **i**), parabrachial nucleus (**j**), locus coeruleus (LC; **k**), and solitary nucleus (Sol; **l**). The dashed line marks the boundaries between brain regions **(d)**. Scale bars = 200 μm in **(a–d,f–l);** 100 μm in **(e)**.

### Summary of Brain-Wide Afferent Inputs to the Striatum in Tree Shrews

**FIGURE 11:**
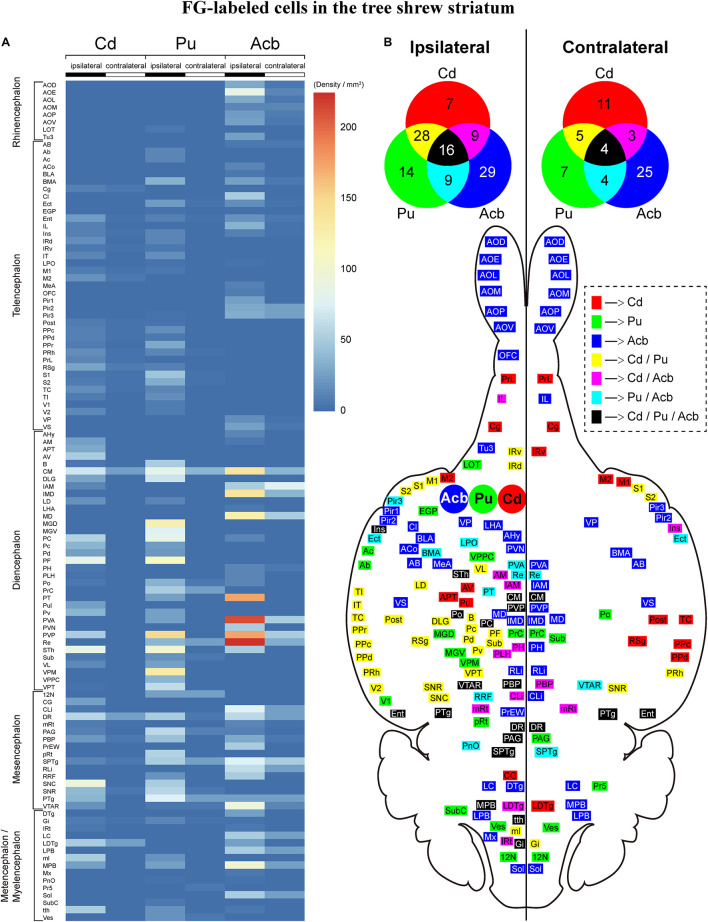
**(A)** Heatmap showing the density of FG-labeled cells projecting to the caudate nucleus (Cd), putamen (Pu), or accumbens nucleus (Acb) in ipsilateral and contralateral brain regions of tree shrews from rostral to caudal. The color of each box represents the density of FG-labeled cells in each brain region of the rhinencephalon, telencephalon, diencephalon, mesencephalon, metencephalon, and myelencephalon. The color bar indicates the range of density values, with the strongest density shown in red and the weakest density in blue. **(B)** The Cd-, Pu-, and/or Acb-projecting regions in the ipsilateral and contralateral brain hemispheres are displayed on the horizontal section of the tree shrew brain. The injection sites of the FG retrograde tracer in the Cd (red), Pu (green), and Acb (blue) were located in the left brain hemisphere. Venn diagrams showing the number and overlap of the Cd-, Pu-, and/or Acb-projecting regions in the ipsilateral and contralateral brain hemispheres. Color squares in the dashed box indicate the brain regions in the horizontal section and Venn diagrams which project to the Cd, Pu, and/or Acb. The numbers in the Venn diagrams show the number of the brain regions which project to the Cd, Pu, and/or Acb. For abbreviations, see Abbreviations.

Overall, we compared the whole-brain input patterns of the striatal subregions in tree shrews (Figure 11A). The main inputs to the Cd are from the CM, PC, PF, STh, DR, SNC, and LDTg. The major inputs to the Pu originate from the S1, CM, DLG, MGD, MGV, PC, PF, PVP, STh, and VPM. The majority of Acb-projecting neurons are located in the AOE, CM, IMD, MD, PT, PVA, PVP, Re, VTAR, and MPB. Generally, the number of ipsilateral labeled neurons was larger than the number of contralateral labeled neurons throughout the tree shrew brain with FG injected into the Cd, Pu, and Acb (Figure 11A). The Cd, Pu, and Acb received inputs from different neuronal populations in the ipsilateral (60, 67, and 63 brain regions, respectively) and contralateral (23, 20, and 36 brain regions, respectively) brain regions (Figure 11). Moreover, the Cd and Pu received inputs from the same 44 ipsilateral and 9 contralateral regions (Figure 11B). The Cd and Acb received afferent projections from 25 ipsilateral and 7 contralateral common brain regions (Figure 11B). The Acb and Pu were innervated by inputs from the same 25 ipsilateral and 8 contralateral regions (Figure 11B). Additionally, the Cd, Pu, and Acb received afferent projections from the same 16 ipsilateral and 4 contralateral structures (Figure 11B).

## Discussion

The cytoarchitecture and chemoarchitecture of the Cd, ic, Pu, and Acb in tree shrews were investigated in this study. In addition, we compared the distributive features of PV, NOS, CR, and TH in the striatum of tree shrews with those of rats. Our data showed distinct distribution patterns of these proteins between the tree shrew and rat striatum. Finally, we mapped the distribution of whole-brain input neurons projecting to the Cd, Pu, and Acb of tree shrews using a retrograde tracing strategy. We discovered that the neurons projecting to the Cd, Pu, and Acb exhibited distinct distribution characteristics across the whole brain. Comprehensive comparisons of whole-brain inputs to the Cd, Pu, and Acb of tree shrews are made with those of other animals including rodents, cats, dogs, and monkeys (Supplementary Table 1).

### Cytoarchitecture and Chemoarchitecture of the Striatum

In primates and humans, the striatum is divided into a ventral striatum and a dorsal striatum (Vo et al., 2014; Marche et al., 2017; Ketchesin et al., 2021). The ventral striatum contains the Acb. The dorsal striatum consists of the Cd and Pu. In rodents, same papers also support that the striatum has dorsal and ventral subdivisions (Voorn et al., 2004; Ferré et al., 2010). In addition, there are many papers that explicitly list the Acb and striatum as separate structures in rats (Missale et al., 1985; Cass et al., 1992; Zhou et al., 2004; Miller et al., 2012). Previous data have indicated that the tree shrew is closer to primates than to rodents in terms of brain evolution (Sillitoe et al., 2004; Rice et al., 2011; Ni et al., 2015, 2018; Römer et al., 2018; Maher et al., 2021). Therefore, the architectonic subdivisions of the striatum in the tree shrew are delineated according to previous works in primates and humans (Vo et al., 2014; Marche et al., 2017; Ketchesin et al., 2021).

Interestingly, we observed that the well-developed ic in the dorsal striatum of tree shrews clearly separated the Pu and Cd, which is in accordance with previous reports (Rice et al., 2011; Ni et al., 2018). The present study found that PV, NOS, CR, and TH had a wide distribution within the striatum of tree shrews and rats. This set of antibodies was chosen according to a previous work on neurochemical characterization of the tree shrew dorsal striatum (Rice et al., 2011). Although the distribution of PV-ir neurons and fibers in the striatum has been investigated previously (Rice et al., 2011; Ni et al., 2018), our study revealed that the density of PV-ir neurons was different in the distinct subregions of the tree shrew striatum and similar in the various parts of the rat striatum. Additionally, patch-like (striosome) areas were more prominent in the tree shrew dorsal striatum than in the rat dorsal striatum. A previous immunohistochemical study showed that the distribution of striosomes detected by immunohistochemistry for calbindin, acetylcholinesterase, and TH in the tree shrew dorsal striatum was similar to that reported in primates (Rice et al., 2011). There was a higher density of NOS-ir neurons in the ventral striatum than in the dorsal striatum of tree shrews and rats. Moreover, a higher density of CR-ir neurons was observed in the ventral striatum than in the dorsal striatum of tree shrews but not in that of rats. Interestingly, densely and intensely stained CR-ir fibers were present in the dorsal but not the ventral part of the ic in tree shrews, which was different from the results observed in rats. A comparison of the distribution of CR-ir cells in the striatum has revealed that CR is located in the most abundant type of interneurons in humans and monkeys, whereas the most abundant striatal interneurons in rats are those expressing PV (Wu and Parent, 2000). The small- and medium-sized CR-ir neurons are the most abundant class of striatal interneurons in humans (Petryszyn et al., 2014). About 80% of the large-sized CR-ir neurons in striatum is colocalized with choline acetyltransferase in humans that are not found in rodents (Cicchetti et al., 1998; Petryszyn et al., 2014). Furthermore, we observed no significant differences in the density of TH-ir fibers between the dorsal and ventral striatum of tree shrews and rats. In the tree shrew striatum, the ic contained the lowest density of PV-, NOS-, CR-, and TH-ir cells and fibers. Our data show that the density of PV-, NOS-, CR-, and TH-ir cells or fibers in the Cd, Pu, and Acb of tree shrews is higher than that in rats. The cytoarchitectonics of the striatum in tree shrews has a closer resemblance to that of primates than rodents. The highly developed striatum in tree shrews may play an important role in the locomotion ability of climbing, jumping, and leaping in arboreal habitats.

The PV-, NOS-, CR-, and TH-ir neurons in the striatum play crucial roles in the regulation of motor and non-motor functions (Calabresi et al., 2000; Smeets et al., 2000). The selective deficit of striatal PV interneurons in Tourette syndrome subjects may result in impaired regulation of neuron firing in the striatum (Kataoka et al., 2010). Previous evidence indicates that the administration of a NOS inhibitor (7-nitroindazole) dose-dependently induces a marked motor deficit in mice (Araki et al., 2001). Surprisingly, the NOS inhibitor also attenuates abnormal and excessive movements induced by repeated administration of L-DOPA in rats (Padovan-Neto et al., 2009). Recent studies indicate that CR interneuron density in the caudate nucleus is lower in individuals with autism spectrum disorder and in patients with schizophrenia (Adorjan et al., 2017, 2020). The loss of striatal TH interneurons in TH-Cre mice induced by chemogenetic techniques impairs instrumental goal-directed behavior while preserving motoric and appetitive behaviors (Kaminer et al., 2019). When a defective herpes simplex virus type 1 vector expressing human TH was delivered into the striatum of 6-hydroxydopamine–lesioned rats used as a model of Parkinson’s disease, long-lasting expression of TH and long-term behavioral recovery were maintained for 1 year (During et al., 1994). However, further experiments are needed to confirm the functions of PV, NOS, CR, and TH in the Cd, Pu, and Acb of tree shrews.

### Brain-Wide Afferent Projections to the Caudate Nucleus

Our data indicate that the Cd in the tree shrew brain receives afferent inputs from the telencephalon, diencephalon, mesencephalon, metencephalon, and myelencephalon. In the telencephalon, the Cd receives afferent inputs that mainly originate from the RSg, M2, and Ent, which is similar to the results reported in other mammals (Finch, 1996; Guo et al., 2015). It is known that inefficient connectivity between the Cd and cerebral cortex potentially accounts for stereotypic behaviors, sensorimotor skill learning, and executive impairments in autism (Turner et al., 2006). Moreover, skilled forelimb use and tongue-forelimb synchronization depend upon the neural organization between the Cd and motor cortex in rats (Whishaw et al., 1986; Zhuravin et al., 1994). In the diencephalon of tree shrews, the major inputs to the Cd originate from the STh, PF, CM, AV, and PVP, which is in agreement with previous reports in other animals (Royce, 1978; de las Heras et al., 1999; Guo et al., 2015). A previous study demonstrated that dopamine release in the Cd and Pu of rhesus macaques depends upon the precise location of the stimulation site in the STh (Min et al., 2016). In addition, abnormal networks between the Cd and the thalamus are involved in Alzheimer’s disease, Huntington’s disease, depression, and schizophrenia pathogenesis (Aron et al., 2003; Ryan et al., 2013; Torres-Platas et al., 2016). In the mesencephalon of tree shrews, the majority of inputs to the Cd arise from the DR, SNC, and SNR, which is consistent with previous studies in cats (Royce, 1978). Previous results indicate that excitatory amino acids in the DR are involved in the regulation of striatal serotonin release in rats (Kalén et al., 1989). High-frequency stimulation of the SNC alters dopamine content and causes a reversible decrease in responses in the Cd (Yeoman et al., 1981). In rats, the concentrations of dopamine in the Cd is increased by grafting fetal substantia nigra to the Cd (Freed et al., 1980). Additionally, the Cd in the tree shrew brain receives sparse to moderate afferent inputs from other brain regions, including the APT, B, CLi, CG, DLG, Pd, Ent, Gi, IT, IRd, infraradiata ventral area (IRv), IAM, IRt, LDTg, MPB, mRt, PBP, PLH, PTg, PH, PPc, posterior parietal dorsal area (PPd), PPr, postsubiculum (Post), Re, Sub, SPTg, TC, TI, Pv, and VL. These Cd-projecting regions in tree shrews are not found in other mammals. However, the functions of the neural circuits between the Cd and these brain regions are still unknown.

### Brain-Wide Afferent Projections to the Putamen

Our findings indicate that the Pu in tree shrews receives whole-brain afferent inputs from the forebrain (rhinencephalon, telencephalon, and diencephalon), midbrain (mesencephalon), and hindbrain (metencephalon and myelencephalon). In the rhinencephalon and telencephalon, the majority of direct synaptic inputs to the Pu originate from the S1 and S2, which is also found in the monkey brain (Künzle, 1977; Graziano and Gross, 1993). Previous investigation indicates that more time spent playing online games predicts greater functional connectivity between the Pu and S1 in adolescents with internet gaming disorder and lower functional connectivity between the two regions in healthy controls (Hong et al., 2015). In the diencephalon of tree shrews, the majority of the external input neurons are located in the CM, VPM, STh, PVP, PF, and PC, which is in agreement with previous reports in other mammals (Rasminsky et al., 1973; Parent et al., 1983; Smith and Parent, 1986; Hsu and Price, 2009; Guo et al., 2015). Previous results suggest that the PF is capable of facilitating dopaminergic neurotransmission in the caudate-putamen (Kilpatrick and Phillipson, 1986). Abnormal functional connectivity between the Pu and the cortical-striatal-thalamic circuits may underlie the pathological basis of attention deficit hyperactivity disorder (Cao et al., 2009). In addition, the Pu and thalamus form a coherent corticosubcortical anatomical network associated with reading, speech production, and spatial neglect in humans (Karnath et al., 2002; Seghier and Price, 2010). In the midbrain and hindbrain of tree shrews, Pu-projecting neurons originate mainly from the SNC and SNR, which is similar to previous findings in other animals (Smith and Parent, 1986). A recent study suggests that rats with unilateral 6-hydroxydopamine substantia nigra lesions show enhanced locomotor and stereotyped behaviors (Chang et al., 2021). Furthermore, Parkinson’s disease is characterized by the loss of dopaminergic neurons from the substantia nigra that project to the caudate-putamen (Xicoy et al., 2020). In addition to the Pu-projecting regions mentioned above, the Pu-projecting neurons in tree shrews are also located in the BMA, B, DLG, Ent, Gi, 12N, IT, IRd, IRv, LPO, LD, MGD, MGV, MPB, LOT, pRt, PBP, PT, PTg, PAG, PnO, Post, PrC, RRF, SubC, Sub, SPTg, TC, TI, VPPC, VTAR, and Ves. To the best of our knowledge, these Pu-projecting brain regions have not been reported in other species. Further studies are needed to investigate the functions of the neural circuits between the Pu and these input regions.

### Brain-Wide Afferent Projections to the Accumbens Nucleus

In the present study, our results suggest that the Acb receives brain-wide afferent inputs from the rhinencephalon, telencephalon, diencephalon, mesencephalon, metencephalon, and myelencephalon. In the rhinencephalon and telencephalon, Acb-projecting neurons mainly originate from the anterior olfactory nucleus, Cl, IL, BMA, and VS in tree shrews, which is in agreement with previous results in mice (Li et al., 2018; Ma et al., 2020). According to previous findings, the IL-Acb pathway plays a critical role in cocaine seeking, ethanol-induced behavior, decision-making modality, and social recognition (Sweis et al., 2018; Bechard et al., 2021; Ferreira et al., 2021; Park et al., 2021). In addition, previous findings highlight a role for the VS-Acb pathway in cocaine seeking, schizophrenia, and stress susceptibility (McGlinchey et al., 2016; Lee et al., 2019; Saoud et al., 2020). In the diencephalon of tree shrews, the majority of afferent inputs to the Acb arise from the PVT, Re, PT, MD, IMD, and CM, which is in line with previous studies in mice (Li et al., 2018; Ma et al., 2020). Recent studies indicate an important role for the PVT-Acb pathway in heroin seeking, food seeking, stress-induced social avoidance, novelty-suppressed feeding, and reward behavior (Cheng et al., 2018; Choudhary et al., 2018; Meffre et al., 2019; Dong et al., 2020; Chisholm et al., 2021). Additionally, MD glutamatergic synapses to medium spiny neurons in the Acb are likely a substrate for motivation and reward learning (Turner et al., 2018). In the mesencephalon, metencephalon, and myelencephalon of tree shrews, afferent inputs to the Acb originate largely from the DR, VTAR, and MPB, which is in accord with previous reports in mice (Li et al., 2018; Ma et al., 2020). Earlier research has confirmed the role of the DR-Acb pathway in prosocial interactions, cocaine seeking, and alcohol-drinking behavior (Yoshimoto et al., 2012; Dölen et al., 2013; Kasper et al., 2016; Walsh et al., 2018). Moreover, recent findings suggest that the VTAR-Acb pathway plays an important role in cocaine seeking, feeding, anesthesia, sexual motivation, attention, and impulsive behavior (Addy et al., 2018; Cortes et al., 2019; Bond et al., 2020; Flores-Dourojeanni et al., 2021; Gui et al., 2021). Additionally, the Acb in tree shrews receives sparse to moderate extrastriatal inputs from other brain areas, such as the DTg, Gi, IRt, Tu3, matrix region of the medulla (Mx), PLH, Po, PrEW, and SPTg, which is different from the observations in other mammals. However, the functions of the neural circuits between the Acb and these brain areas in tree shrews are not yet well understood.

In the present study, we provide fundamental data to compare the brain-wide input patterns of the striatum in tree shrews. The Cd and Pu receive the majority of their inputs from the same upstream brain regions, indicating that they may integrate the same information from these upstream regions and be involved in regulating the same physiological functions and behaviors. The Cd- and Pu-projecting neurons in tree shrews are also present in different upstream brain regions, suggesting different roles in motor-related behaviors (Yoshida et al., 1991; Brovelli et al., 2011). Although the role of the caudate-putamen nucleus in rodents has been widely investigated in previous studies (Szczypka et al., 2001; Charntikov et al., 2017; Wang et al., 2021), how the Cd and Pu regulate physiological functions and behaviors through their own pathways remains unknown. The dorsal striatum (Cd and Pu) and ventral striatum (Acb) receive the majority of inputs from distinct upstream brain regions in tree shrews, indicating that they have different roles in the modulation of motor and non-motor functions (Costall et al., 1977; Nomoto et al., 2000). However, the diverse roles of different projection patterns in the Cd, Pu, and Acb are still poorly understood.

**TABLE 1:** Comparison of thalamic inputs into the striatal subregions of tree shrews (present study) with those of other animals (for detailed comparison of whole-brain inputs and complete data, see Supplementary Table 1).

Brain region	Tree shrew			Rodent		Other references		
	Cd	Pu	Acb	CPu^1^	Acb^2, 3^	Cd	Pu	Acb
Anterior pretectal nucleus (APT)	✓				✓			
Anteromedial thalamic nucleus (AM)	✓		✓	✓		✓ (monkey^4^)	✓ (monkey^5^)	✓ (rat^6^)
Anteroventral thalamic nucleus (AV)	✓					✓ (cat^7^)		
Brachium of the superior colliculus (B)	✓	✓						
Central medial thalamic nucleus (CM)	✓	✓	✓		✓	✓ (cat^7^)	✓ (cat^8^)	
Central nucleus of the pulvinar (Pc)	✓	✓				✓ (monkey^9^)	✓ (monkey^9^)	
Dorsal lateral geniculate nucleus (DLG)	✓	✓						
Dorsal nucleus of the pulvinar (Pd)	✓	✓					✓ (monkey^9^)	
Interanteromedial thalamic nucleus (IAM)	✓		✓		✓			
Intermediodorsal thalamic nucleus (IMD)			✓		✓			
Laterodorsal thalamic nucleus (LD)	✓	✓				✓ (cat^7^)		
Medial geniculate nucleus, dorsal part (MGD)		✓						
Medial geniculate nucleus, ventral part (MGV)		✓						
Mediodorsal thalamic nucleus (MD)			✓	✓	✓	✓ (cat^7^)	✓ (monkey^5^)	
Paracentral thalamic nucleus (PC)	✓	✓	✓	✓	✓	✓ (cat^7^)	✓ (monkey^5^)	
Parafascicular thalamic nucleus (PF)	✓	✓		✓	✓	✓ (cat^7^)		
Paratenial thalamic nucleus (PT)		✓	✓		✓			✓ (monkey^4^)
Paraventricular thalamic nucleus (PVT), anterior part (PVA)		✓	✓		✓	✓ (cat^10^)	✓ (monkey^4^)	✓ (monkey^4^)
Paraventricular thalamic nucleus, posterior part (PVP)	✓	✓	✓		✓	✓ (cat^10^)		
Posterior complex of the thalamus (Po)	✓	✓	✓	✓		✓ (cat^11^)		
Postsubiculum (Post)	✓	✓						
Pulvinar nuclei (Pul)	✓	✓				✓ (monkey^9^)	✓ (monkey^9^)	
Reuniens thalamic nucleus (Re)	✓	✓	✓		✓		✓ (monkey^4^)	✓ (monkey^4^)
Submedius thalamic nucleus (Sub)	✓	✓						
Ventral nucleus of the pulvinar (Pv)	✓	✓					✓ (monkey^9^)	
Ventral posterior nucleus of the thalamus, parvicellular part (VPPC)		✓						
Ventral posterior thalamic nucleus (VPT)	✓	✓		✓				
Ventral posteromedial thalamic nucleus (VPM)		✓		✓			✓ (monkey^9^)	
Ventral tegmental area, rostral part (VTAR)	✓	✓	✓		✓	✓ (cat^7^)		
Ventrolateral thalamic nucleus (VL)	✓	✓					✓ (monkey^9^)	

*^1^Guo et al., 2015; ^2^Li et al., 2018; ^3^Ma et al., 2020; ^4^Hsu and Price, 2009; ^5^Smith and Parent, 1986; ^6^Christie et al., 1987; ^7^Royce, 1978; ^8^Rasminsky et al., 1973; ^9^Parent et al., 1983; ^10^de las Heras et al., 1999; ^11^Jayaraman, 1985.*

There are several limitations to our study. First, retrograde FG is widely used to mark projecting cell bodies (Mondello et al., 2016; Lanciego and Wouterlood, 2020), but it cannot provide the detailed morphology of labeled neurons and fibers. Second, it can be difficult to inject FG into an entire striatal subregion without spilling over to the adjacent areas. To prevent FG diffusion along the micropipette track and spreading to adjacent areas, we injected a small volume of FG into the striatal subregion very slowly. Third, various subpopulations of Cd-, Pu-, and Acb-projecting neurons within the same upstream brain regions have not been identified in tree shrews. Use of another retrograde tracer to compare extent of label would be useful in establishing the validity of the findings. In the future, different projection neurons within the upstream regions can be examined using a double-labeling method of cholera toxin subunit B conjugated with Alexa Fluor 488 and 555 (Ma et al., 2020). In addition, new genetic and Cre-dependent viral approaches will be needed to examine the diverse cell types in the tree shrew striatum with higher specificity than fluorescent dye alone. Fourth, the calbindin expression, colocalization among these proteins, colocalization of FG with dopamine receptors in tree shrews should be investigated in future study. Moreover, cell types in the striatum express PV, NOS, CR, and TH should be identified.

In summary, the present results provide a comprehensive comparison of the cytoarchitecture and chemoarchitecture of the Cd, ic, Pu, and Acb in tree shrews and rats by using immunohistochemical detection of PV, NOS, CR, and TH. Moreover, our data show the distinct distributions of PV-, NOS-, CR-, and TH-ir neurons and/or fibers in the dorsal and ventral striatum between tree shrews and rats. In addition, the current paper presents whole-brain maps of afferent inputs to the Cd, Pu, and Acb in tree shrews. Our findings demonstrate that the Cd, Pu, and Acb receive afferent inputs from the same and different upstream brain regions in tree shrews. Taken together, our new findings support the tree shrew as an alternative model for investigating neural circuits in striatal subregions and human striatal disorders.

## Data Availability Statement

The raw data supporting the conclusions of this article will be made available by the authors, without undue reservation.

## Ethics Statement

The animal study was reviewed and approved by the Animal Care and Use Committees of the Sichuan University and the University of Science and Technology of China.

## Author Contributions

R-JN was involved in experimental treatment of the animal, acquisition of data, histochemical analysis, interpretation of data, and writing the manuscript. Y-MS was involved in experimental treatment of the animal and tissue processing. TL was responsible for revising manuscript. J-NZ was responsible for experiment designing and revising manuscript. All the authors approved the final manuscript.

## Conflict of Interest

The authors declare that the research was conducted in the absence of any commercial or financial relationships that could be construed as a potential conflict of interest.

## Publisher’s Note

All claims expressed in this article are solely those of the authors and do not necessarily represent those of their affiliated organizations, or those of the publisher, the editors and the reviewers. Any product that may be evaluated in this article, or claim that may be made by its manufacturer, is not guaranteed or endorsed by the publisher.

## Glossary

2, layer 2 of cortex; 3, layer 3 of cortex; 10N, dorsal motor nucleus of vagus; 12n, root of hypoglossal nerve; 12N, hypoglossal nucleus; 3N, oculomotor nucleus; 3PC, oculomotor nucleus, parvicellular part; 3V, 3rd ventricle; 4n, trochlear nerve; 4N, trochlear nucleus; 4V, 4th ventricle; 5N, motor trigeminal nucleus; 6n, root of abducens nerve; 6N, abducens nucleus; 7N, facial nucleus; 7n, facial nerve; 8cn, cochlear root of the vestibulocochlear nerve; 8vn, vestibular root of the vestibulocochlear nerve; A5, A5 noradrenaline cells; AA, anterior amygdaloid area; Ab, auditory belt area; AB, accessory basal nucleus; Ac, auditory core area; ac, anterior commissure; Acb, accumbens nucleus; AcbC, accumbens nucleus, core; AcbSh, accumbens nucleus, shell; aci, anterior commissure, intrabulbar part; ACo, anterior cortical amygdaloid nucleus; acp, anterior commissure, posterior part; AD, anterodorsal thalamic nucleus; AHi, amygdalohippocampal area; AHy, anterior hypothalamic area; alv, alveus of the hippocampus; AM, anteromedial thalamic nucleus; Amb, ambiguus nucleus; AmbC, ambiguus nucleus, compact part; AOD, anterior olfactory nucleus, dorsal part; AOE, anterior olfactory nucleus, external part; AOL, anterior olfactory nucleus, lateral part; AOM, anterior olfactory nucleus, medial part; AON, anterior olfactory nucleus; AOP, anterior olfactory nucleus, posterior part; AOV, anterior olfactory nucleus, ventral part, AP, area postrema; APT, anterior pretectal nucleus, APTD, anterior pretectal nucleus, dorsal part; APTV, anterior pretectal nucleus, ventral part; Aq, aqueduct; Arc, arcuate hypothalamic nucleus; ArcP, arcuate hypothalamic nucleus, posterior part; asc7, ascending fibers of the facial nerve; ASt, amygdalostriatal transition area; AV, anteroventral thalamic nucleus; AVPe, anteroventral periventricular nucleus; B, brachium of the superior colliculus; BLA, basolateral amygdaloid nucleus; BMA, basomedial amygdaloid nucleus; BN, basal nucleus (Meynert); CA1, field CA1 of the hippocampus; CA2, field CA2 of the hippocampus; CA3, field CA2 of the hippocampus; cc, corpus callosum; CC, central canal; Cd, caudate nucleus; CeA, central amygdaloid nucleus; Cg, cingulate cortex; CG, central gray; Cl, claustrum; CLi, caudal linear nucleus of the raphe; CM, central medial thalamic nucleus; CnF, cuneiform nucleus; cp, cerebral peduncle; CR, calretinin; csc, commissure of the superior colliculus; Cu, cuneate nucleus; cu, cuneate fasciculus; D3V, dorsal 3rd ventricle; das, dorsal acoustic stria; DCN, dorsal cochlear nucleus; DFC, dorsal frontal cortex; DG, dentate gyrus of the hippocampus; DLG, dorsal lateral geniculate nucleus; DMH, dorsomedial hypothalamic nucleus; DP, dorsal peduncular cortex; DpG, deep gray layer of the superior colliculus; DpWh, deep white layer of the superior colliculus; DR, dorsal raphe nucleus; DS, dorsal subiculum; dsc, dorsal spinocerebellar tract; DTg, dorsal tegmental nucleus; dtgx, dorsal tegmental decussation; E, ependyma and subependymal layer; EA, sublenticular extended amygdala; EAC, sublenticular extended amygdala, central part; Ect, ectorhinal cortex; ECu, external cuneate nucleus; EGP, external globus pallidus; Ent, entorhinal cortex; EPl, external plexiform layer of the olfactory bulb; EPlA, external plexiform layer of the accessory olfactory bulb; EW, Edinger-Westphal nucleus; f, fornix; FG, Fluoro-Gold; fi, fimbria of the hippocampus; fmi, forceps minor of the corpus callosum; fr, fasciculus retroflexus; g7; genu of the facial nerve; GCL, granule cell layer of the olfactory bulb; Gem, gemini hypothalamic nucleus; Gi, gigantocellular reticular nucleus; GL, glomerular layer of the olfactory bulb; GlA, glomerular layer of the accessory olfactory bulb; GP, globus pallidus; Gr, gracile nucleus; GrA, granule cell layer of the accessory olfactory bulb; GrDG, granular layer of the dentate gyrus; HDB, nucleus of the horizontal limb of the diagonal band; Hip, hippocampus; IAM, interanteromedial thalamic nucleus; ic, internal capsule; IC, inferior colliculus; ICj, islands of Calleja; icp, inferior cerebellar peduncle; IF, interfascicular nucleus; IG, indusium griseum; IGL, intergeniculate leaf; IGP, internal globus pallidus; IL, infralimbic cortex; IM, intercalated amygdaloid nucleus, main part; IMD, intermediodorsal thalamic nucleus; InC, interstitial nucleus of Cajal; InG, intermediate gray layer of the superior colliculus; Ins, insular cortex; Int, interposed cerebellar nucleus; InWh, intermediate white layer of the superior colliculus; IO, inferior olive; IP, interpeduncular nucleus; IPA, interpeduncular nucleus, apical subnucleus; IPAC, interstitial nucleus of the posterior limb of the anterior commissure; IPC, interpeduncular nucleus, caudal subnucleus; IPl, internal plexiform layer of the olfactory bulb; IPL, interpeduncular nucleus, lateral subnucleus; IPR, interpeduncular nucleus, rostral subnucleus; IRd, infraradiata dorsalis; IRt, intermediate reticular nucleus; IRv, infraradiata ventral area; IT, inferior temporal cortex; JPLH, juxtaparaventricular part of lateral hypothalamus; Lat, lateral cerebellar nucleus; LC, locus coeruleus; LD, laterodorsal thalamic nucleus; LDTg, laterodorsal tegmental nucleus; lfp, longitudinal fasciculus of the pons; LHA, lateral hypothalamic area; LHb, lateral habenular nucleus; LHbL, lateral habenular nucleus, lateral part; LHbM, lateral habenular nucleus, medial part; LL, nucleus of the lateral lemniscus; ll, lateral lemniscus; LM, lateral mammillary nucleus; LMoL, lacunosum moleculare layer of the hippocampus; lo, lateral olfactory tract; LOT, nucleus of the lateral olfactory tract; LPB, lateral parabrachial nucleus; LPO, lateral preoptic area; LRt, lateral reticular nucleus; LSd, lateral septal nucleus, dorsal part; LSi, lateral septal nucleus, intermediate part; LSS, lateral stripe of the striatum; LSv, lateral septal nucleus, ventral part; LV, lateral ventricle; M1, primary motor cortex; M2, secondary motor cortex; m5, motor root of the trigeminal nerve; MCL, mitral cell layer of the olfactory bulb; mcp, middle cerebellar peduncle; MD, mediodorsal thalamic nucleus; MdD, medullary reticular nucleus, dorsal part; MdV, medullary reticular nucleus, ventral part; ME, median eminence; me5, mesencephalic trigeminal tract; Me5, mesencephalic trigeminal nucleus; MeA, medial amygdaloid nucleus, Med, medial cerebellar nucleus; MFC, medial frontal cortex; MG, medial geniculate nucleus; MGD, medial geniculate nucleus, dorsal part; MGV, medial geniculate nucleus, ventral part; MHb, medial habenular nucleus; MiA, mitral cell layer of the accessory olfactory bulb; MiTg, microcellular tegmental nucleus; ml, medial lemniscus; ML, medial mammillary nucleus, lateral part; mlf, medial longitudinal fasciculus; mlx, medial lemniscus decussation; MM, medial mammillary nucleus, medial part; MnPO, median preoptic nucleus; MnR, median raphe nucleus; MoDG, molecular layer of the dentate gyrus; MPA, medial preoptic area; MPB, medial parabrachial nucleus; MPO, medial preoptic nucleus; MPT, medial pretectal nucleus; MRe, mammillary recess of the 3rd ventricle; mRt, mesencephalic reticular formation; MS, medial septal nucleus; mt, mammillothalamic tract; MT, medial terminal nucleus of the accessory optic tract; Mx, matrix region of the medulla; NDB, nucleus of the diagonal band; NOS, nitric oxide synthase; oc, olivocerebellar tract; oc/dsc, olivocerebellar tract and dorsal spinocerebellar tract; och, optic chiasm; OFC, orbital frontal cortex; ONL, olfactory nerve layer; Op, optic nerve layer of the superior colliculus; opt, optic tract; OPT, olivary pretectal nucleus; Or, oriens layer of the hippocampus; OV, olfactory ventricle (olfactory part of lateral ventricle); P5, peritrigeminal zone; PaA, paraventricular hypothalamic nucleus, anterior part; PAG, periaqueductal gray; PaP, paraventricular hypothalamic nucleus, posterior part; PaV, paraventricular hypothalamic nucleus, ventral part; PBG, parabigeminal nucleus; PBP, parabrachial pigmented nucleus of the VTA; PC, paracentral thalamic nucleus, Pc, central nucleus of the pulvinar; pc, posterior commissure; PCRt, parvicellular reticular nucleus; Pd, dorsal nucleus of the pulvinar; Pe, periventricular hypothalamic nucleus; PeF, perifornical nucleus; PF, parafascicular thalamic nucleus; PG, pineal gland; PH, posterior hypothalamic nucleus; PHA, posterior hypothalamic area; Pir1, piriform cortex, layer 1; PLH, peduncular part of lateral hypothalamus; PMD, premammillary nucleus, dorsal part; PMV, premammillary nucleus, ventral part; Pn, pontine nuclei; PN, paranigral nucleus of the VTA; PnC, pontine reticular nucleus, caudal part; PnO, pontine reticular nucleus, oral part; Po, posterior complex of the thalamus; PoDG, polymorph layer of the dentate gyrus; PoMn, posteromedian thalamic nucleus; Post, postsubiculum; pPAG, p1 periaqueductal gray; PPc, posterior parietal caudal area; PPd, posterior parietal dorsal area; PPr, posterior parietal rostral area; Pr, prepositus nucleus; Pr5, principal sensory trigeminal nucleus; PrC, precommissural nucleus; PrEW, pre-Edinger-Westphal nucleus; PRh, perirhinal cortex; PrL, prelimbic cortex; PrS, presubiculum; pRt, p1 reticular formation; PT, paratenial thalamic nucleus; PTg, pedunculopontine tegmental nucleus; Pu, putamen; Pul, pulvinar nuclei; Pv, ventral nucleus of the pulvinar; PV, parvalbumin; PvA, parietal ventral area; PVA, paraventricular thalamic nucleus, anterior part; PVN, paraventricular hypothalamic nucleus; PVP, paraventricular thalamic nucleus, posterior part; py, pyramidal tract; Py, pyramidal cell layer of the hippocampus; pyx, pyramidal decussation; Rad, radiatum layer of the hippocampus; Rbd, rhabdoid nucleus; Re, reuniens thalamic nucleus; Rh, rhomboid thalamic nucleus; RLi, rostral linear nucleus of the raphe; RMC, red nucleus, magnocellular part; RN, red nucleus; Ro, nucleus of Roller; RPa, raphe pallidus nucleus; RPC, red nucleus, parvicellular part; RRF, retrorubral field; rs, rubrospinal tract; RSg, retrosplenial granular cortex; Rt, reticular thalamic nucleus; RtTg, reticulotegmental nucleus of the pons; S1, primary somatosensory cortex; S2, secondary somatosensory cortex; s5, sensory root of the trigeminal nerve; SC, superior colliculus; SCC, somatosensory caudal cortex; SCN, suprachiasmatic nucleus; scp, superior cerebellar peduncle; scpd, superior cerebellar peduncle, descending limb; SFi, septofimbrial nucleus; SFO, subfornical organ; SGe, supragenual nucleus; SHi, septohippocampal nucleus; SHy, septohypothalamic nucleus; sm, stria medullaris of the thalamus; SNC, substantia nigra, compact part; SNL, substantia nigra, lateral part; SNR, substantia nigra, reticular part; SO, superior olive; Sol, solitary nucleus; SON, supraoptic nucleus; Sp5, spinal trigeminal nucleus; sp5, spinal trigeminal tract; SPa, subparaventricular zone of the hypothalamus; SPTg, subpeduncular tegmental nucleus; SpVe, spinal vestibular nucleus; st, stria terminalis; STh, subthalamic nucleus; StHy, striohypothalamic nucleus; STL, bed nucleus of the stria terminalis, lateral division; STMA, bed nucleus of the stria terminalis, medial division, anterior part; STMAL, bed nucleus of the stria terminalis, medial division, anterolateral part; STMAM, bed nucleus of the stria terminalis, medial division, anteromedial part; STMP, bed nucleus of the stria terminalis, medial division, posterior part; STMPL, bed nucleus of the stria terminalis, medial division, posterolateral part; STMPM, bed nucleus of the stria terminalis, medial division, posteromedial part; STMV, bed nucleus of the stria terminalis, medial division, ventral part; STr, subiculum, transition area; str, superior thalamic radiation; Su5, supratrigeminal nucleus; Sub, submedius thalamic nucleus; SubC, subcoeruleus nucleus; SuG, superficial gray layer of the superior colliculus; SuM, supramammillary nucleus; SuML, supramammillary nucleus, lateral part; SuMM, supramammillary nucleus, medial part; sumx, supramammillary decussation; SuVe, superior vestibular nucleus; TC, temporal cortex; tfp, transverse fibers of the pons; TH, tyrosine hydroxylase; TI, temporal inferior area; TS, triangular septal nucleus; tth, trigeminothalamic tract; Tu, olfactory tubercle; Tu1, layer 1 of olfactory tubercle; TuLH, tuberal region of lateral hypothalamus; tz, trapezoid body; Tz, nucleus of the trapezoid body; V1, primary visual cortex; V2, secondary visual cortex; VCA, ventral cochlear nucleus, anterior part; VCN, ventral cochlear nucleus; VDB, nucleus of the vertical limb of the diagonal band; VeCb, vestibulocerebellar nucleus; Ves, vestibular nucleus; VL, ventrolateral thalamic nucleus; VLG, ventral lateral geniculate nucleus; VLN, ventrolateral reticular nucleus; VM, ventromedial thalamic nucleus; VMH, ventromedial hypothalamic nucleus; VP, ventral pallidum; VPM, ventral posteromedial thalamic nucleus; VPO, ventral periolivary nucleus; VPPC, ventral posterior nucleus of the thalamus, parvicellular part; VPT, ventral posterior thalamic nucleus; VS, ventral subiculum; vsc, ventral spinocerebellar tract; VTA, ventral tegmental area; VTAR, ventral tegmental area, rostral part; VTg, ventral tegmental nucleus; vtgx, ventral tegmental decussation; VTM, ventral tuberomammillary nucleus; xscp, decussation of the superior cerebellar peduncle; ZI, zona incerta; Zo, zonal layer of the superior colliculus.
