# Supplementary material for: Whole-Brain Afferent Inputs to the Caudate Nucleus, Putamen, and Accumbens Nucleus in the Tree Shrew Striatum
Source: Front Neuroanat. 2021 Nov 2;15:763298. doi: 10.3389/fnana.2021.763298 (PMC8593333; doi:10.3389/fnana.2021.763298)
Supplement: Supplementary file 1 [file Table_1.docx]

**Supplementary Table 1.** Comparison of whole-brain inputs into the striatal subregions of tree shrews (present study) with those of other animals.

| **Brain region** | **Tree shrew** | | | **Rodent** | | **Other references** | | |
| --- | --- | --- | --- | --- | --- | --- | --- | --- |
|  | **Cd** | **Pu** | **Acb** | **CPu**  (Guo et al., 2015) | **Acb**  (Li et al., 2018; Ma et al., 2020) | **Cd** | **Pu** | **Acb** |
| accessory basal nucleus (AB) |  |  | 🗸 |  |  |  |  | 🗸(rat; (Wright et al., 1996)) |
| anterior cortical amygdaloid nucleus (ACo) |  | 🗸 | 🗸 |  | 🗸 |  | 🗸 (cat; (Gorbachevskaya, 1990)) |  |
| anterior hypothalamic area (AHy) |  |  | 🗸 |  | 🗸 |  |  |  |
| anterior olfactory nucleus, dorsal part (AOD) |  |  | 🗸 |  | 🗸 |  |  |  |
| anterior olfactory nucleus, external part (AOE) |  |  | 🗸 |  |  |  |  |  |
| anterior olfactory nucleus, lateral part (AOL) |  |  | 🗸 |  |  |  |  |  |
| anterior olfactory nucleus, medial part (AOM) |  |  | 🗸 |  |  |  |  |  |
| anterior olfactory nucleus, posterior part (AOP) |  |  | 🗸 |  |  |  |  |  |
| anterior olfactory nucleus, ventral part (AOV) |  |  | 🗸 |  |  |  |  |  |
| anterior pretectal nucleus (APT) | 🗸 |  |  |  | 🗸 |  |  |  |
| anteromedial thalamic nucleus (AM) | 🗸 |  | 🗸 | 🗸 |  | 🗸 (monkey; (Hsu and Price, 2009)) | 🗸 (monkey; (Smith and Parent, 1986)) | 🗸 (rat; (Christie et al., 1987)) |
| anteroventral thalamic nucleus (AV) | 🗸 |  |  |  |  | 🗸 (cat; (Royce, 1978)) |  |  |
| auditory belt area (Ab) |  | 🗸 |  | 🗸 | 🗸 |  | 🗸 (cat; (Zhao et al., 2015)) |  |
| auditory core area (Ac) |  | 🗸 |  |  |  |  |  |  |
| basolateral amygdaloid nucleus (BLA) |  | 🗸 | 🗸 | 🗸 | 🗸 | 🗸 (cat; (Jayaraman, 1985)) | 🗸 (monkey; (Parent et al., 1983)) |  |
| basomedial amygdaloid nucleus (BMA) |  | 🗸 | 🗸 |  | 🗸 | 🗸 (cat; (Royce, 1978)) |  |  |
| brachium of the superior colliculus (B) | 🗸 | 🗸 |  |  |  |  |  |  |
| caudal linear nucleus of the raphe (CLi) | 🗸 |  | 🗸 |  |  |  |  | 🗸 (rat; (Brog et al., 1993)) |
| central gray (CG) | 🗸 |  |  |  |  |  |  |  |
| central medial thalamic nucleus (CM) | 🗸 | 🗸 | 🗸 |  | 🗸 | 🗸 (cat; (Royce, 1978)) | 🗸 (cat; (Rasminsky et al., 1973)) |  |
| central nucleus of the pulvinar (Pc) | 🗸 | 🗸 |  |  |  | 🗸 (monkey; (Parent et al., 1983)) | 🗸 (monkey; (Parent et al., 1983)) |  |
| cingulate cortex (Cg) | 🗸 |  |  | 🗸 | 🗸 | 🗸 (cat; (Rosell and Giménez-Amaya, 1999)) |  |  |
| claustrum (Cl) |  |  | 🗸 |  | 🗸 |  |  |  |
| dorsal lateral geniculate nucleus (DLG) | 🗸 | 🗸 |  |  |  |  |  |  |
| dorsal nucleus of the pulvinar (Pd) | 🗸 | 🗸 |  |  |  |  | 🗸 (monkey; (Parent et al., 1983)) |  |
| dorsal raphe nucleus (DR) | 🗸 | 🗸 | 🗸 | 🗸 | 🗸 | 🗸 (cat; (Royce, 1978)) | 🗸 (monkey; (Parent et al., 1983)) |  |
| dorsal tegmental nucleus (DTg) |  |  | 🗸 |  |  |  |  |  |
| ectorhinal cortex (Ect) |  | 🗸 | 🗸 | 🗸 | 🗸 |  |  |  |
| entorhinal cortex (Ent) | 🗸 | 🗸 | 🗸 |  | 🗸 | 🗸 (rat; (Finch, 1996)) | |  |
| external globus pallidus (EGP) |  | 🗸 |  | 🗸 |  |  | 🗸 (monkey; (Parent et al., 1983)) |  |
| gigantocellular reticular nucleus (Gi) | 🗸 | 🗸 | 🗸 |  |  |  |  |  |
| hypoglossal nucleus (12N) |  | 🗸 |  |  |  |  |  |  |
| inferior temporal cortex (IT) | 🗸 | 🗸 |  |  |  |  |  |  |
| infralimbic cortex (IL) | 🗸 |  | 🗸 |  | 🗸 | 🗸 (cat; (Rosell and Giménez-Amaya, 1999)) |  |  |
| infraradiata dorsalis (IRd) | 🗸 | 🗸 |  |  |  |  |  |  |
| infraradiata ventral area (IRv) | 🗸 | 🗸 |  |  |  |  |  |  |
| insular cortex (Ins) | 🗸 | 🗸 | 🗸 |  | 🗸 | 🗸 (cat; (Rosell and Giménez-Amaya, 1999)) | 🗸 (monkey; (Parent et al., 1983)) |  |
| interanteromedial thalamic nucleus (IAM) | 🗸 |  | 🗸 |  | 🗸 |  |  |  |
| intermediate reticular nucleus (IRt) | 🗸 |  | 🗸 |  |  |  |  |  |
| intermediodorsal thalamic nucleus (IMD) |  |  | 🗸 |  | 🗸 |  |  |  |
| lateral hypothalamic area (LHA) |  |  | 🗸 |  | 🗸 |  |  |  |
| lateral parabrachial nucleus (LPB) |  |  | 🗸 |  | 🗸 |  |  |  |
| lateral preoptic area (LPO) |  | 🗸 | 🗸 |  | 🗸 |  |  |  |
| laterodorsal tegmental nucleus (LDTg) | 🗸 |  | 🗸 |  |  |  |  | 🗸 (rat; (Brog et al., 1993)) |
| laterodorsal thalamic nucleus (LD) | 🗸 | 🗸 |  |  |  | 🗸 (cat; (Royce, 1978)) |  |  |
| layer 3 of olfactory tubercle (Tu3) |  |  | 🗸 |  |  |  |  |  |
| locus coeruleus (LC) |  |  | 🗸 |  | 🗸 |  |  |  |
| matrix region of the medulla (Mx) |  |  | 🗸 |  |  |  |  |  |
| medial amygdaloid nucleus (MeA) |  |  | 🗸 |  | 🗸 |  |  |  |
| medial geniculate nucleus, dorsal part (MGD) |  | 🗸 |  |  |  |  |  |  |
| medial geniculate nucleus, ventral part (MGV) |  | 🗸 |  |  |  |  |  |  |
| medial lemniscus (ml) | 🗸 | 🗸 |  |  |  | 🗸 (cat; (Vandermaelen et al., 1978)) |  |  |
| medial parabrachial nucleus (MPB) | 🗸 | 🗸 | 🗸 |  | 🗸 |  |  |  |
| mediodorsal thalamic nucleus (MD) |  |  | 🗸 | 🗸 | 🗸 | 🗸 (cat; (Royce, 1978)) | 🗸 (monkey; (Smith and Parent, 1986)) |  |
| mesencephalic reticular formation (mRt) | 🗸 |  | 🗸 |  | 🗸 |  |  |  |
| nucleus of the lateral olfactory tract (LOT) |  | 🗸 |  |  |  |  |  |  |
| orbital frontal cortex (OFC) |  |  | 🗸 | 🗸 | 🗸 |  |  |  |
| p1 reticular formation (pRt) |  | 🗸 |  |  |  |  |  |  |
| parabrachial pigmented nucleus of the ventral tegmental area (PBP) | 🗸 | 🗸 | 🗸 |  |  |  |  | 🗸 (rat; (Hasue and Shammah-Lagnado, 2002)) |
| paracentral thalamic nucleus (PC) | 🗸 | 🗸 | 🗸 | 🗸 | 🗸 | 🗸 (cat; (Royce, 1978)) | 🗸 (monkey; (Smith and Parent, 1986)) |  |
| parafascicular thalamic nucleus (PF) | 🗸 | 🗸 |  | 🗸 | 🗸 | 🗸 (cat; (Royce, 1978)) |  |  |
| paratenial thalamic nucleus (PT) |  | 🗸 | 🗸 |  | 🗸 |  |  | 🗸 (monkey; (Hsu and Price, 2009)) |
| paraventricular hypothalamic nucleus (PVN) |  |  | 🗸 |  | 🗸 |  |  |  |
| paraventricular thalamic nucleus (PVT), anterior part (PVA) |  | 🗸 | 🗸 |  | 🗸 | 🗸 (cat; (de las Heras et al., 1999)) | 🗸 (monkey; (Hsu and Price, 2009)) | 🗸 (monkey; (Hsu and Price, 2009)) |
| paraventricular thalamic nucleus, posterior part (PVP) | 🗸 | 🗸 | 🗸 |  | 🗸 | 🗸 (cat; (de las Heras et al., 1999)) |  |  |
| peduncular part of lateral hypothalamus (PLH) | 🗸 |  | 🗸 |  |  |  |  |  |
| pedunculopontine tegmental nucleus (PTg) | 🗸 | 🗸 | 🗸 |  | 🗸 |  |  |  |
| periaqueductal gray (PAG) | 🗸 | 🗸 | 🗸 |  | 🗸 | 🗸 (cat; (Royce, 1983)) |  |  |
| perirhinal cortex (PRh) | 🗸 | 🗸 | 🗸 | 🗸 | 🗸 |  | 🗸 (dog; (Chivileva, 1996)) |  |
| piriform cortex, layer 1 (Pir1) |  |  | 🗸 | 🗸 | 🗸 |  |  |  |
| piriform cortex, layer 2 (Pir2) |  |  | 🗸 |  | 🗸 |  |  |  |
| piriform cortex, layer 3 (Pir3) |  | 🗸 | 🗸 |  | 🗸 |  |  |  |
| pontine reticular nucleus, oral part (PnO) |  | 🗸 | 🗸 |  | 🗸 |  |  |  |
| posterior complex of the thalamus (Po) | 🗸 | 🗸 | 🗸 | 🗸 |  | 🗸 (cat; (Jayaraman, 1985)) |  |  |
| posterior hypothalamic nucleus (PH) | 🗸 |  | 🗸 |  | 🗸 |  |  |  |
| posterior parietal caudal area (PPc) | 🗸 | 🗸 |  |  |  |  | 🗸 (monkey; (Cavada and Goldman-Rakic, 1991)) |  |
| posterior parietal dorsal area (PPd) | 🗸 | 🗸 |  |  |  |  |  |  |
| posterior parietal rostral area (PPr) | 🗸 | 🗸 |  |  |  |  |  |  |
| postsubiculum (Post) | 🗸 | 🗸 |  |  |  |  |  |  |
| precommissural nucleus (PrC) |  | 🗸 |  |  |  |  |  |  |
| pre-Edinger-Westphal nucleus (PrEW) |  |  | 🗸 |  |  |  |  |  |
| prelimbic cortex (PrL) | 🗸 |  |  | 🗸 | 🗸 | 🗸 (cat; (Rosell and Giménez-Amaya, 1999)) |  |  |
| primary motor cortex (M1) | 🗸 | 🗸 |  | 🗸 |  | 🗸 (cat; (Rosell and Giménez-Amaya, 1999)) | 🗸 (monkey; (Künzle, 1975)) |  |
| primary somatosensory cortex (S1) | 🗸 | 🗸 |  | 🗸 |  | 🗸 (cat; (Rosell and Giménez-Amaya, 1999)) | 🗸 (monkey; (Künzle, 1977)) |  |
| primary visual cortex (V1) |  | 🗸 |  | 🗸 |  |  |  |  |
| principal sensory trigeminal nucleus (Pr5) |  |  |  |  |  |  |  |  |
| pulvinar nuclei (Pul) | 🗸 | 🗸 |  |  |  | 🗸 (monkey; (Parent et al., 1983)) | 🗸 (monkey; (Parent et al., 1983)) |  |
| retrorubral field (RRF) |  | 🗸 | 🗸 |  | 🗸 | 🗸 (cat; (Royce, 1978)) |  |  |
| retrosplenial granular cortex (RSg) | 🗸 | 🗸 |  | 🗸 |  |  |  |  |
| reuniens thalamic nucleus (Re) | 🗸 | 🗸 | 🗸 |  | 🗸 |  | 🗸 (monkey; (Hsu and Price, 2009)) | 🗸 (monkey; (Hsu and Price, 2009)) |
| rostral linear nucleus of the raphe (RLi) |  |  | 🗸 |  | 🗸 |  |  |  |
| secondary motor cortex (M2) | 🗸 |  |  | 🗸 |  |  |  |  |
| secondary somatosensory cortex (S2) | 🗸 | 🗸 |  | 🗸 |  |  | 🗸 (monkey; (Graziano and Gross, 1993)) |  |
| secondary visual cortex (V2) | 🗸 | 🗸 |  | 🗸 |  |  |  |  |
| solitary nucleus (Sol) |  |  | 🗸 |  |  |  |  | 🗸 (rat; (Brog et al., 1993)) |
| subcoeruleus nucleus (SubC) |  | 🗸 |  |  |  |  |  |  |
| submedius thalamic nucleus (Sub) | 🗸 | 🗸 |  |  |  |  |  |  |
| subpeduncular tegmental nucleus (SPTg) | 🗸 | 🗸 | 🗸 |  |  |  |  |  |
| substantia nigra, compact part (SNC) | 🗸 | 🗸 |  | 🗸 |  | 🗸 (cat; (Royce, 1978)) | 🗸 (monkey; (Smith and Parent, 1986)) |  |
| substantia nigra, reticular part (SNR) | 🗸 | 🗸 |  |  |  | 🗸 (cat; (Royce, 1978)) | 🗸 (monkey; (Smith and Parent, 1986)) |  |
| subthalamic nucleus (STh) | 🗸 | 🗸 | 🗸 | 🗸 | 🗸 |  |  |  |
| temporal cortex (TC) | 🗸 | 🗸 |  |  | 🗸 |  |  |  |
| temporal inferior area (TI) | 🗸 | 🗸 |  |  |  |  |  |  |
| trigeminothalamic tract (tth) | 🗸 | 🗸 | 🗸 |  |  |  |  |  |
| ventral nucleus of the pulvinar (Pv) | 🗸 | 🗸 |  |  |  |  | 🗸 (monkey; (Parent et al., 1983)) |  |
| ventral pallidum (VP) |  |  | 🗸 |  | 🗸 |  |  |  |
| ventral posterior nucleus of the thalamus, parvicellular part (VPPC) |  | 🗸 |  |  |  |  |  |  |
| ventral posterior thalamic nucleus (VPT) | 🗸 | 🗸 |  | 🗸 |  |  |  |  |
| ventral posteromedial thalamic nucleus (VPM) |  | 🗸 |  | 🗸 |  |  | 🗸 (monkey; (Parent et al., 1983)) |  |
| ventral subiculum (VS) |  |  | 🗸 |  | 🗸 |  |  |  |
| ventral tegmental area, rostral part (VTAR) | 🗸 | 🗸 | 🗸 |  | 🗸 | 🗸 (cat; (Royce, 1978)) |  |  |
| ventrolateral thalamic nucleus (VL) | 🗸 | 🗸 |  |  |  |  | 🗸 (monkey; (Parent et al., 1983)) |  |
| vestibular nucleus (Ves) |  | 🗸 |  |  |  |  |  |  |
